# Phylogeny and diversity of *Rigidoporus* (*Hymenochaetales*, *Basidiomycota*), including three new species from Asia

**DOI:** 10.3389/fcimb.2023.1216277

**Published:** 2023-06-19

**Authors:** Chao-Ge Wang, Josef Vlasák, Can Jin, Jing Si

**Affiliations:** ^1^ Institute of Microbiology, School of Ecology and Nature Conservation, Beijing Forestry University, Beijing, China; ^2^ Biology Centre, Institute of Plant Molecular Biology, Czech Academy of Sciences, České Budějovice, Czechia

**Keywords:** *Oxyporaceae*, polypore, taxonomy, wood-decaying fungi, phylogeny

## Abstract

Phylogenetic and morphological analyses on *Rigidoporus* were carried out. The genus *Rigidoporus* (*Hymenochaetales*, *Basidiomycota*), typified by *R. microporus* (Fr.) Overeem. (synonym *Polyporus micromegas* Mont.), was established by Murrill in 1905. The genus is mainly characterized by annual to perennial, resupinate, effused-reflexed to pileate or stipitate basidiomata with azonate or concentrically zonate and sulcate upper surface, a monomitic to pseudo-dimitic hyphal structure, simple-septate generative hyphae, and ellipsoid to globose basidiospores. Phylogeny on species of the genus is reconstructed with two loci DNA sequences including the internal transcribed spacer regions and the large subunit. Three new species in *Rigidoporus* are described and illustrated from Asia, and one new combination in the genus is proposed. The main morphological characteristics of the currently accepted species of *Rigidoporus* are provided.

## 1 Introduction

The genus *Rigidoporus* Murrill (*Hymenochaetales*, *Basidiomycota*), typified by *Rigidoporus microporus* (Fr.) Overeem. (synonym *Polyporus micromegas* Mont.), was established by Murrill (1905). This type of species is an important parasite on cultivated tropical trees, widely distributed in the tropical zone (Ryvarden and Melo, 2017). Traditionally, the genus is characterized by a fawn, reddish to dark tube layer, which is very hard in dried material contrasting with corky to waxy consistency of the context, a monomitic or pseudo-dimitic hyphal system with simple-septate, often in part strongly sclerified generative hyphae, ovoid to globose basidiospores, inamyloid and indextrinoid walls of hyphae and spores, and causing a white rot (Murrill, 1905; Pouzar, 1966; Ryvarden and Melo, 2017).

Later, *Rigidoporus* was often recognized as a confusing genus with *Oxyporus* (Bourdot & Galzin) Donk, which was established by Donk (1933) for species with similar hyphae and spores but tubes of waxy consistency. Pouzar (1966) treated *Oxyporus* as a subgenus of *Rigidoporus.* However, light-colored basidiomata with thick-walled, encrusted, hymenial cystidia are mostly present in species of *Oxyporus*, while basidiomata with colored pore surface, thick-walled encrusted hyphoid cystidia, and mammillate cystidioles are mostly in *Rigidoporus* (Ryvarden and Melo, 2017).

*Rigidoporus* is also morphologically similar to *Physisporinus* P. Karst., but this similarity is evidently of only a superficial character. Many taxa had been described in *Rigidoporus* according to morphology (Ryvarden, 1972a; Ryvarden, 1972b; Ryvarden, 1983; Dai, 1998; Núñez and Ryvarden, 1999; Buchanan and Ryvarden, 2000; Núñez et al., 2001; Vampola and Vlasák, 2012; Ryvarden, 2015), but phylogenetic studies demonstrated that they should be classified into *Physisporinus* belonging to *Polyporales* (Wu et al., 2017).

Wu et al. (2017) revealed that *Rigidoporus* belongs to *Hymenochaetales* Oberw., and *Oxyporus* was considered as a synonym of *Rigidoporus*. Currently, *Rigidoporus* and *Oxyporus* are merged as one genus based on phylogenetic and morphological studies and absorbed into *Oxyporaceae* Zmitr. & Malysheva (Zmitrovich and Malysheva, 2014; Wu et al., 2017). Then, Wang et al. (2023) reactivated *Rigidoporaceae* Jülich and *Oxyporaceae* as its synonyms. However, only 14 species have been accepted in *Rigidoporus* based on phylogenetic analyses until now (Wu et al., 2017; Yuan et al., 2020), and many lack molecular data. In this study, a comprehensive study about *Rigidoporus* is displayed including phylogenetic and morphological analyses. Phylogeny based on a two-gene dataset (ITS + nLSU) is carried out. Three new species that occur in Asia in the genus are described and illustrated, and one new combination in the genus is proposed. The main morphological characteristics of the currently accepted species of *Rigidoporus* are provided.

## 2 Materials and methods

### 2.1 Morphological studies

The studied specimens are deposited in the fungoria of the Institute of Microbiology, Beijing Forestry University (BJFC) and the Institute of Applied Ecology, Chinese Academy of Sciences (IFP), Museum Vysociny Jihlava, Czech Republic (MJ), herbarium of V.N. Karazin National University, Kharkiv, Ukraine (CWU), the private fungorium of Josef Vlasák (JV), which will be later deposited at the National Museum Prague of Czech Republic (PRM). Morphological descriptions are conducted based on field notes and fungoria specimens. The microscopic analysis refers to Miettinen et al. (2018) and Wu et al. (2022). Sections were studied at a magnification of up to ×1,000 using a Nikon Eclipse 80i microscope and phase contrast illumination. Microscopic features and measurements were made from slide preparations stained with Cotton Blue and Melzer’s reagent. Spores were measured from sections cut from the tubes. To represent variations in the size of spores, 5% of measurements were excluded from each end of the range and are given in parentheses. In the description, KOH = 5% potassium hydroxide, IKI = Melzer’s reagent, IKI− = neither amyloid nor dextrinoid, CB = Cotton Blue, CB+ = cyanophilous in Cotton Blue, CB− = acyanophilous in Cotton Blue, L = arithmetic average of spore length, W = arithmetic average of spore width, Q = L/W ratios, and n = number of basidiospores/measured from given number of specimens. Color terms are recognized from Anonymous (1969) and Petersen (1996).

### 2.2 DNA extraction, amplification, and sequencing

A Hexadecyl trimethyl ammonium bromide (CTAB) rapid plant genome extraction kit-DN14 (Aidlab Biotechnologies Co., Ltd, Beijing) was used to obtain DNA from dried specimens and to perform the polymerase chain reaction (PCR) according to the manufacturer’s instructions with some modifications (Li et al., 2014; Shen et al., 2019). Two DNA gene fragments, i.e., internal transcribed spacer (ITS) and large subunit nuclear ribosomal RNA gene (nLSU), were amplified using the primer pairs ITS5/ITS4 and LR0R/LR7 (White et al., 1990; Hopple and Vilgalys, 1999) (http://www.biology.duke.edu/fungi/mycolab/primers.htm). The PCR procedure for ITS was as follows: initial denaturation at 95°C for 3 min, followed by 34 cycles of denaturation at 94°C for 40 s, annealing at 54°C for 45 s, and extension at 72°C for 1 min, and a final extension of 72°C for 10 min. The PCR procedure for nLSU was as follows: initial denaturation at 94°C for 1 min, followed by 34 cycles of denaturation at 94°C for 30 s, annealing at 50°C for 1 min, and extension at 72°C for 1.5 min, and a final extension at 72°C for 10 min. All sequences analyzed in this study are listed in **Table 1**.

**Table 1:** Taxa information and GenBank accession numbers of the sequences used in this study.

Species name	Sample no.	Location	GenBank accession No.	
			ITS	nLSU
*Bjerkandera adusta*	Dai 15665	China: Yunnan	MW507098	MW520205
*Bjerkandera fulgida*	Dai 21193	Malaysia	OQ930240^a^	OQ924520^a^
*Bridgeoporus nobilissimus*	SP-K	USA	AF508346	—
*Bridgeoporus nobilissimus*	RN-B	USA	AF508338	—
*Bridgeoporus sinensis*	Dai 11367	China: Jilin	KY131833	KY131892
*Bridgeoporus sinensis*	Cui 13490	China: Jilin	OQ930241^a^	—
*Exidiopsis calcea*	KHL 11075	Sweden	AY463406	AY586654
*Flaviporus minutus*	Dai 21167	Malaysia	OQ930242^a^	OQ924521^a^
*Flaviporus minutus*	Dai 16222	China: Hainan	KY131881	KY131938
*Flaviporus minutus*	Dai 21164	Malaysia	OQ930243^a^	OQ924522^a^
*Flavodon subulatus*	Dai 13102	China: Yunnan	OQ930244^a^	OQ924523^a^
*Flavodon subulatus*	Dai 13143	China: Yunnan	OQ930245^a^	OQ924524^a^
*Hyphoderma litschaueri*	FP-101740-Sp	USA	KP135295	KP135219
*Hyphoderma setigerum*	FD 312	USA	KP135297	KP135222
*Hyphodontia abieticola*	5181b	Sweden	DQ873601	DQ873601
*Hyphodontia floccosa*	3069b	Sweden	DQ873618	DQ873618
*Hyphodontia paradoxa*	FCUG 2425	Russia	AF145571	AY059067
*Hyphodontia radula*	PDD 91616	New Zealand	GQ411525	AJ406466
*Irpex laceratus*	Dai 11682	China: Hunan	OQ930246^a^	—
*Irpex laceratus*	Dai 13393	China: Zhejiang	OQ930247^a^	OQ924525^a^
*Leifia brevispora*	LWZ 20170820-46	China	MK343469	MK343473
*Leifia flabelliradiata*	KG Nilsson 36270	Sweden	DQ873635	DQ873635
*Leifia* sp.	LWZ 20171015-36	Viet Nam	MK343471	MK343475
*Leifia* sp.	Cui 13659	China: Hainan	OQ930248^a^	—
*Leifia* sp.	Dai 12013	China: Hainan	OQ930249^a^	—
*Leucophellinus hobsonii*	Cui 6468	China: Hainan	KT203288	KT203309
*Leucophellinus irpicoides*	Dai 8277	China: Jilin	KY131841	KY131900
*Leucophellinus irpicoides*	Dai 6356	China: Zhejiang	KY131840	KY131899
*Meruliopsis nanlingensis*	Dai 8173	China: Hunan	JX623942	JX644053
*Phanerina mellea*	Dai 19585	Sri Lanka	OQ930250^a^	OQ924526^a^
*Phanerina mellea*	Dai 19592	Sri Lanka	OQ930251^a^	OQ924527^a^
*Phellinotus neoaridus*	URM 80362	Brazil	NR158813	—
*Phellinus ferrugineovelutinus*	Cui 10042	China: Jilin	KC782527	KC782529
*Physisporinus* sp.	Dai 19793	China: Yunnan	OM669891	OM669975
*Physisporinus eminens*	Dai 11400	China: Jilin	KY131852	KY131909
*Physisporinus eminens*	Dai 20832	China: Jilin	MT279689	MT279689
*Physisporinus sanguinolentus*	Dai 20995	Belarus	MT309483	—
*Physisporinus sanguinolentus*	Dai 21030	Belarus	MT309482	—
*Rigidoporus aurantiacus*	JV 2106/103-J	Ecuador	OQ941871^a^	—
*Rigidoporus aurantiacus*	JV 1906/M8	Ecuador	OQ941872^a^	—
*Rigidoporus corticola*	Dai 12632	Finland	KF111018	KF111020
*Rigidoporus corticola*	Dai 15941	China: Xinjiang	OQ930252^a^	OQ924528^a^
*Rigidoporus corticola*	Cui 9862	China: Heilongjiang	OQ930253^a^	OQ924529^a^
*Rigidoporus cuneatus*	Dai 6404	China: Zhejiang	KY131876	KY131932
*Rigidoporus cuneatus*	Cui 10855	China: Sichuan	OQ930254^a^	OQ924530^a^
*Rigidoporus cuneatus*	Cui 10857	China: Sichuan	OQ930255^a^	OQ924531^a^
*Rigidoporus cuneatus*	Dai 24344	China: Guizhou	OQ930256^a^	OQ924532^a^
*Rigidoporus ginkgonis*	Cui 5558	China: Beijing	KT203296	KT203317
*Rigidoporus ginkgonis*	Dai 24460	China: Beijing	OQ930257^a^	OQ924533^a^
*Rigidoporus ginkgonis*	Dai 1330	China: Beijing	MT309490	MT309473
*Rigidoporus ginkgonis*	Dai 15789	China: Shandong	OQ930258^a^	OQ924534^a^
***Rigidoporus imbricatus***	Dai 17515	China: Yunnan	OQ930259^a^	OQ924535^a^
***Rigidoporus imbricatus***	Dai 21180	Malaysia	OQ930260^a^	OQ924536^a^
*Rigidoporus juniperinus*	Dai 17100	Uzbekistan	OQ930261^a^	OQ924537^a^
*Rigidoporus juniperinus*	YG 1070	Uzbekistan	MK433641	MK433643
*Rigidoporus juniperinus*	Dai 17101	Uzbekistan	OQ930262^a^	—
*Rigidoporus macroporus*	Dai 4044	China: Sichuan	KT203298	KT203319
*Rigidoporus macroporus*	Dai 4146	China: Sichuan	KY131880	KY131937
*Rigidoporus macroporus*	Dai 24198	China: Gansu	OQ930263^a^	OQ924538^a^
“*Rigidoporus microporus*”	ED 310	Nigeria	KJ559458	KJ559523
“*Rigidoporus microporus*”	N 402	Cameroon	KJ559468	KJ559525
“*Rigidoporus microporus*”	FRIM 646	Malaysia	HQ400709	—
“*Rigidoporus microporus*”	taxon 219653	Indonesia	AB697722	—
*Rigidoporus microporus*	Dai 17402	Brazil	OQ930264^a^	—
*Rigidoporus microporus*	Dai 17392	Brazil	OQ930265^a^	—
*Rigidoporus microporus*	JV 2110/1	Ecuador	OQ930266^a^	—
*Rigidoporus millavensis*	Dai 18970	China: Gansu	OQ930267^a^	OQ924539^a^
*Rigidoporus millavensis*	Wei 1622	China: Xinjiang	KT203300	KT203321
*Rigidoporus millavensis*	Dai 24509	China: Inner Mongolia	OQ930268^a^	OQ924540^a^
*Rigidoporus millavensis*	Dai 24503	China: Inner Mongolia	OQ930269^a^	OQ924541^a^
*Rigidoporus obducens*	Dai 6451	China: Zhejiang	KY131884	KY131941
*Rigidoporus obducens*	Dai 11898	China: Anhui	OQ930271^a^	OQ924543^a^
*Rigidoporus obducens*	Dai 18458	China: Jiangsu	OQ930270^a^	OQ924542^a^
*Rigidoporus philadelphi*	Dai 24219	China: Gansu	OQ930272^a^	—
*Rigidoporus philadelphi*	Dai 24218	China: Gansu	OQ930273^a^	OQ924544^a^
*Rigidoporus piceicola*	Dai 12793	USA	KF111019	KF111021
*Rigidoporus piceicola*	Dai 5033	China: Qinghai	KT203301	KT203322
*Rigidoporus populinus*	Dai 12664	Finland	KT203303	KT203324
*Rigidoporus populinus*	Dai 22806	China: Yunnan	OQ930274^a^	OQ924545^a^
**“*Rigidoporus pterocaryae*”**	d1	China	KC414238	—
***Rigidoporus pterocaryae***	Cui 4195	China: Fujian	KY131890	KY131947
***Rigidoporus subcorticola***	Dai 24419	China: Beijing	OQ930275^a^	—
***Rigidoporus subcorticola***	Dai 11319	China: Henan	OQ930276^a^	OQ924546^a^
***Rigidoporus subcorticola***	Dai 8895	China: Heilongjiang	KY131875	KY131931
*Rigidoporus submicroporus*	Dai 16682	Thailand	OQ930277^a^	—
*Rigidoporus submicroporus*	Dai 17499	China: Yunnan	OQ930278^a^	OQ924547^a^
*Rigidoporus submicroporus*	Dai 19429	China: Yunnan	OQ930279^a^	—
*Rigidoporus submicroporus*	Cui 12235	China: Tibet	KY131888	KY131945
*Rigidoporus subpopulinus*	Cui 2236	China: Gansu	KT203305	KT203326
*Rigidoporus subpopulinus*	Cui 2240	China: Gansu	KY131889	KY131946
*Rigidoporus subpopulinus*	Dai 24042	China: Qinghai	OQ930280^a^	OQ924548^a^
*Rigidoporus ulmarius*	KM 178999	UK	KJ559446	—
*Rigidoporus ulmarius*	Dai 18490A	China: Hainan	OQ930281^a^	OQ924549^a^
*Rigidoporus ulmarius*	KM 155306	UK	MZ159373	—
*Rigidoporus ulmarius*	JV 2211/H3-J	USA	OQ941873^a^	—
“*Rigidoporus ulmarius*”	JV 1909/17-J	French Guiana	OQ930282^a^	—
“*Rigidoporus ulmarius*”	JV 1504/40	Costa Rica	OQ930283^a^	—
“*Rigidoporus ulmarius*”	JV 1403/5-J	USA	OQ930284^a^	—

^a^Newly generated sequences in this study. Bold = new taxa.

### 2.3 Phylogenetic analyses

In this study, one combined matrix was reconstructed for phylogenetic analysis and a two-gene dataset (ITS + nLSU) was used to determine the phylogenetic position of new species. The sequence alignments and retrieved topologies were deposited in TreeBase (http://www.treebase.org), under accession ID: 30342 (Reviewer access URL: http://purl.org/phylo/treebase/phylows/study/TB2:S30342?x-access-code=32520482eef1e868d55c54893e12e2e3&format=html). Sequences of *Exidiopsis calcea* KHL 11075, obtained from GenBank, were used as the outgroup (Wu et al., 2017). Phylogenetic analyses were carried out using the approaches of Han et al. (2016) and Zhu et al. (2019). Maximum likelihood (ML) and Bayesian inference (BI) analyses were performed based on the two datasets. The best-fit evolutionary model was selected by hierarchical likelihood ratio tests (HLRTs) and Akaike information criterion (AIC) in MrModeltest 2.2 (Nylander, 2004) after scoring 24 models of evolution in PAUP* v.4.0b10 (Swofford, 2002).

Sequences were analyzed using ML with RAxML-HPC2 through the CIPRES Science Gateway (www.phylo.org; Miller et al., 2009). Branch support (BT) for ML analysis was determined by 1,000 bootstrap replicates. Bayesian phylogenetic inference and Bayesian posterior probabilities (BPPs) were computed with MrBayes v.3.1.2 (Ronquist and Huelsenbeck, 2003). Four Markov chains were run for 1,000,000 generations (two-gene dataset) until the split deviation frequency value was less than 0.01, and trees were sampled every 100 generations. The first 25% of the sampled trees were discarded as burn-in, and the remaining ones were used to reconstruct a majority rule consensus and calculate the BPP of the clades. All trees were viewed in FigTree v.1.4.3 (http://tree.bio.ed.ac.uk/software/figtree/). Branches that received bootstrap supports for ML [≥75% (ML-BS)], and BPP (≥0.95 BPP) were considered as significantly supported. The ML bootstrap (ML) ≥ 50% and BBP (BPP) ≥ 0.90 are displayed on topologies from ML analyses, respectively.

## 3 Results

### 3.1 Molecular phylogeny

**Figure 1:**
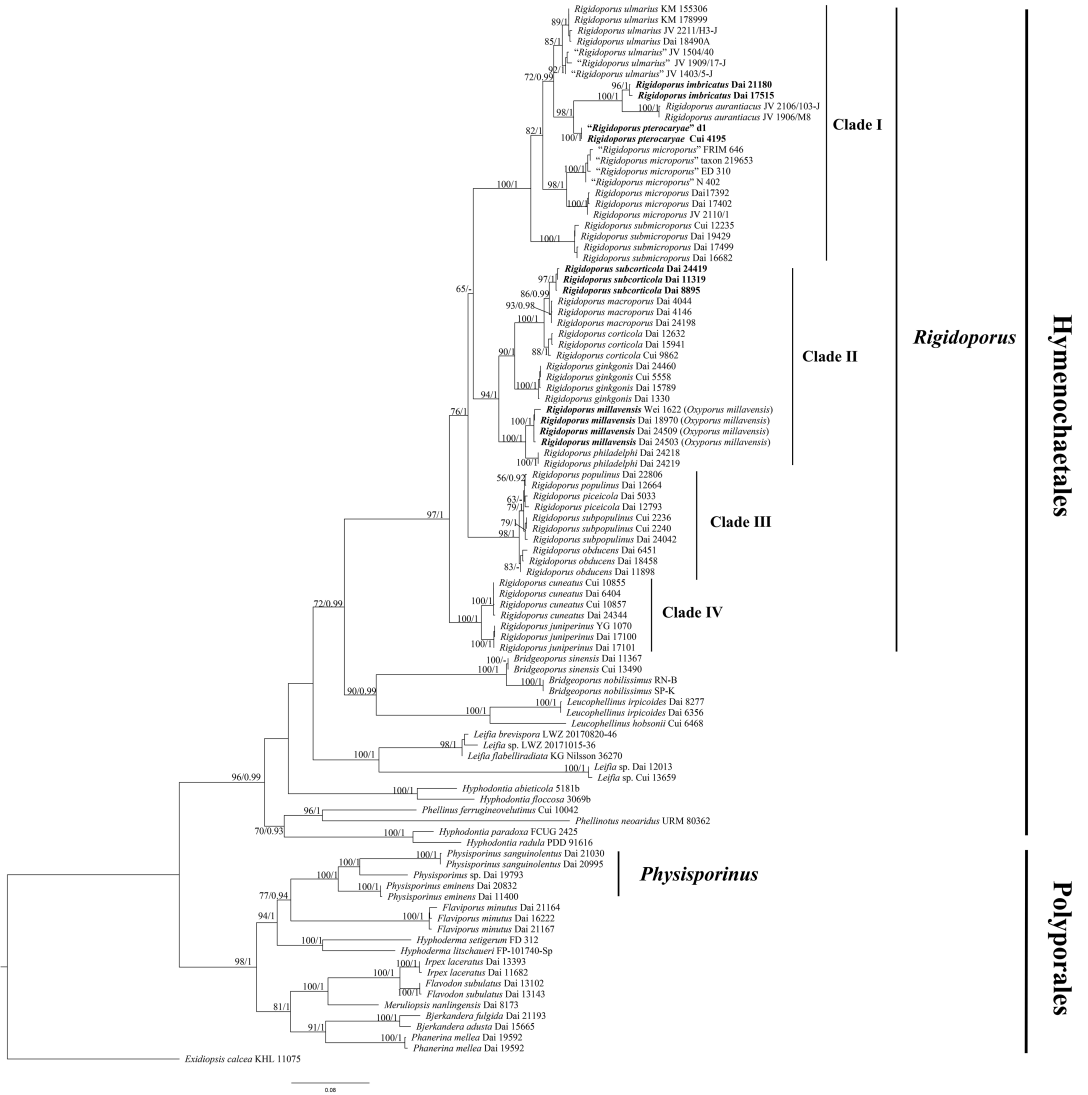
Maximum likelihood (ML) analysis of *Rigidoporus* based on the dataset of ITS + nLSU. ML bootstrap values higher than 50% and Bayesian posterior probability values more than 0.90 are shown. New taxa are in bold.

The combined two-gene dataset (ITS + nLSU) included sequences from 98 samples representing 45 taxa. The dataset had an aligned length of 2,244 characters, of which 1,296 (58%) characters were constant, 174 (8%) were variable and parsimony uninformative, and 774 (34%) were parsimony informative. The phylogenetic reconstruction performed with ML and BI analyses for two combined datasets showed similar topology and few differences in statistical support. The best model-fit applied in the Bayesian analysis was GTR + I + G, lset nst = 6, rates = invgamma, and prset statefreqpr = dirichlet (1, 1, 1, 1). Bayesian analysis resulted in a nearly congruent topology with an average standard deviation of split frequencies as 0.005047 to ML analysis, and thus, only the ML tree is provided (**Figure 1**). The phylogeny reveals that *Rigidoporus* belongs to *Hymenochaetales* and *Physisporinus* belongs to *Polyporales*. There form three independent lineages in *Rigidoporus* as new species.

### 3.2 Taxonomy

***Rigidoporus*** Murrill, Bull. Torrey bot. Club 32(9): 478 (1905) — MycoBank: MB18478

*Type species*. — *Rigidoporus microporus* (Sw.) Overeem, Icon. Fung. Malay. 5: 1 (1924).

*Synonymy*. — *Polyporus micromegas* Mont., Ann. Sci. Nat., Bot., sér. 2 17: 128 (1842).

Basidiomata annual to perennial, resupinate, effused-reflexed to pileate or stipitate, soft to corky when fresh, becoming soft corky to hard when dry. Pileal surface cinnamon to fawn, glabrous or velutinate, azonate or concentrically zonate and sulcate. Pore surface white, fawn, orange to brown black. Hyphal system monomitic to pseudo-dimitic; generative hyphae simple septate, thin- to thick-walled, sometimes subsolid. Hyphoid or hymenial cystidia mostly present, thin- to thick-walled, usually apically encrusted; mammillate cystidioles sometimes present. Basidiospores ovoid, ellipsoid to globose, hyaline, thin- to thick-walled, sometimes with one big guttule, IKI−, CB− or weakly CB+. Causing a white rot.

*Notes*. — *Rigidoporus* is a cosmopolitan genus growth on both angiosperm and gymnosperm woods (Wu et al., 2022). Up to now, 54 taxa in *Rigidoporus* and 18 taxa in *Oxyporus* are recorded in Index Fungorum (http://www.indexfungorum.org/), and among them, 18 species have available DNA sequences (**Table 2**). In this study, three new species are described and illustrated.

**Table 2:** The type locality and main morphological characteristics of species in *Rigidoporus*.

Species	DNA data	Type locality	Shape of basidiomata	Upper surface	Color of poroid surface	Cystidia	Shape of basidiospores	Size of basidiospores (μm)	References
*R. aurantiacus*	+	Venezuela	Annual to perennial, pileate	Orange brown to reddish brown when fresh, pale reddish brown when dry; concentrically zonate and sulcate	Deep orange, fading to ochraceous	—	Globose	3−4	Ryvarden and Iturriaga, 2003
*R. corticola*	+	Finland	Annual to perennial, resupinate to effused-reflexed	White to cream when fresh, pale straw-colored when dry; azonate to slightly zonate, often somewhat radially wrinkled	Cream to pale tan	Thin-walled hymenial cystidia with fine crown crystals; thin-walled gloeocystidia	Ovoid to broadly ellipsoid	5−6 × 3.5−4.5	Ryvarden and Gilbertson, 1994
*R. cuneatus*	+	Canada: British Columbia	Annual, resupinate, effused-reflexed to pileate	White to isabelline; azonate or faintly zonate	White to yellowish	Thin-walled cystidia with capitate crystals	Globose	3−5	Murrill, 1907; Yuan et al., 2020
*R. ginkgonis*	+	China: Beijing	Annual or biennial, resupinate	—	White to cream when fresh, cream to yellowish when dry	—	Broadly ellipsoid to subglobose	5−6 × 4.1−5	Dai and Wang, 2005
***R. imbricatus***	**+**	**China: Yunnan**	**Annual, pileate**	**Yellowish brown to brownish orange when fresh, cinnamon to fawn when dry; concentrically zonate and sulcate, sometimes tuberculate**	**Clay pink to flesh pink when fresh, orange brown when dry**	**—**	**Broadly ellipsoid to subglobose**	**3.4−4 × 2.8−3.2**	**This study**
*R. juniperinus*	+	Uzbekistan	Annual to perennial, resupinate	—	White when fresh, cream to pale ochraceous when dry	Thin-walled hymenial cystidia with crystals; thick-walled hyphoid cystidia with crystals	Broadly ellipsoid to subglobose or globose	4.2−4.5 × 2.9−3	Yuan et al., 2020
*R. macroporus*	+	China: Sichuan	Perennial, resupinate	—	Cream to buff when fresh, pale brownish when dry	Thin- to thick-walled hymenial cystidia with crystals; thin-walled gloeocystidia	Ellipsoid	7−8 × 3.5−4.1	Dai et al., 2004
*R. microporus*	+	Jamaica	Annual to perennial, resupinate, effused-reflexed to pileate	Orange-brown when fresh, wood-colored when dry; concentrically zonate and sulcate	Bright orange to reddish-brown, then pale brown or gray	—	Subglobose	3.5−5 × 3.5−4	Wu et al., 2017
*R. millavensis*	+	France	Annual, resupinate	—	Grayish white to ochraceous	Thin-walled hymenial cystidia with fine crystals or smooth; thin-walled gloeocystidia	Subglobose to globose	4.5−5.5 × 4−4.5	Ryvarden and Melo, 2017
*R. obducens*	+	Germany	Annual, resupinate	—	Pale cream to pale straw-colored	Thick-walled gloeocystidia with crystals	Broadly ellipsoid to subglobose	3−4.5 × 2.5−3.5	Pouzar, 1966
*R. philadelphi*	+	Estonia	Annual, resupinate	—	White to pale cream	Thin-walled hymenial cystidia with fine crystals or smooth; thin-walled gloeocystidia	Subglobose to globose	4.5−5.5 × 4−4.5	Pouzar, 1966
*R. piceicola*	+	China: Qinghai	Annual, resupinate	—	Cream to cinnamon buff when dry	Thin- to thick-walled hyphoid cystidia with crystals	Ellipsoid	4.6−5.3 × 3−3.6	Cui and Dai, 2009
*R. populinus*	+	Sweden	Annual to perennial, resupinate, effused-reflexed to pileate	Whitish, pallid ochraceous, cream or clay-colored; azonate	Ochreaceous to pale ferruginous	Thin- to thick-walled hymenial cystidia with coarse crystals	Broadly ellipsoid to Subglobose	3.5−4.5 × 3−4	Ryvarden and Johansen, 1980
***R. pterocaryae***	**+**	**China: Fujian**	**Annual, pileate**	**Honey yellow when dry; azonate and tuberculate**	**Buff, honey yellow to fawn when dry**	**—**	**Subglobose**	**6.8−7.5 × 5.8−7**	**This study**
***R. subcorticola***	**+**	**China: Beijing**	**Annual, resupinate to effused-reflexed**	**White to cream when fresh, cream to pinkish buff when dry; azonate and tuberculate**	**white to cream when fresh, cream to buff yellow when dry**	**Thick-walled hymenial cystidia with coarse crystals**	**Oblong ellipsoid to ellipsoid**	**5−5.8 × 3−4**	**This study**
*R. submicroporus*	+	China: Tibet	Perennial, resupinate	—	Olivaceous buff when fresh, fawn when dry	Ventricose, thin-walled hymenial cystidia, smooth	Subglobose	3.2−3.7 × 2.8−3.2	Wu et al., 2017
*R. subpopulinus*	+	China: Qinghai	Perennial, effused-reflexed to pileate	White, cream to pale buff; azonate	Cream to pale buff	Thick-walled hyphoid cystidia with coarse crystals	Ellipsoid	3.4−4.7 × 2.3−3.2	Cui et al., 2006
*R. ulmarius*	+	UK	Perennial, resupinate to effused-reflexed	White to cream when fresh, ochraceous to pale tan when dry; azonate, concentrically sulcate, radially wrinkled or irregularly tuberculate	Pinkish salmon to orange brown, then smoky gray	—	Subglobose to globose	6−8 × 5−6.5	Imazeki, 1952
*R. adnatus*	*	Malaysia	Annual, resupinate	—	White to pale ochraceous when fresh, to pinkish ochraceous when dry	hyphoid cystidia with crystals	Ellipsoid	2.5−3.2 × 1.7−2	Corner, 1987
*R. albiporus*	*	Singapore	Annual, resupinate	—	Pale ochraceous	—	Ovoid	4.5−5.5 × 2.8−3.5	Ryvarden and Johansen, 1980; Corner, 1992
*R. amazonicus*	*	Brazil	Annual, laterally stipitate	Ochraceous with some olivaceous tints; azonate and wrinkled	Isabelline	—	Drop-shaped to ellipsoid	4−4.5 × 3−3.5	Ryvarden, 1987
*R. aureofulvus*	*	New Zealand	Annual, pileate	Orange or reddish-orange when fresh, tobacco-brown or black when dry; concentrically zonate or radially striate	Orange-rufous when fresh, reddish brown or dark brick red when dry	—	Subglobose to globose	4.5−6	Buchanan and Ryvarden, 1988
*R. biokoensis*	*	Samoa	Annual, pileate to laterally stipitate	Pale tan, fulvous to dark ochraceous; concentrically zonate and sulcate	Dark ochraceous to pale dirty brown	Thin- to thick-walled hyphoid cystidia with crystals or smooth	Globose	4−4.5	Ryvarden, 1972b
*R. camschadalicus*	*	Russia: Kamchatka	*	*	*	*	*	*	Domanski, 1974
*R. cystidioides*	*	Singapore	Resupinate	—	Bright golden orange, rich cinnamon orange to golden fulvous or lurid ferruginous ochraceous when fresh, lurid mustard yellow when dry	Thick-walled hyphoid cystidia with crystals	Broadly ellipsoid to subglobose	3.5−4.5 × 3−4	Corner, 1987
*R. defibulatus*	*	Ghana	Annual, pileate, laterally stipitate	Pale fuscous ochraceous; concentrically zonate and slightly sulcate	Cream when fresh, light golden brown when dry	—	Subglobose	4−5 × 3.5−4.5	Corner, 1987
*R. dextrinoideus*	*	Kenya	Annual to perennial, resupinate	—	Pale ochraceous, cork-colored to very pale brown with a whitish tint	—	Ellipsoid	3−4.5 × 2−2.5	Johansen and Ryvarden, 1979
*R. dimiticus*	*	Malaysia	Effused-reflexed	White; faintly zonate	Brownish orange	Hyphoid cystidia with crystals	Subglobose	4−5 × 3−4	Hattori, 2001a
*R. erectus*	*	Solomon Islands	Annual, pileate to stipitate	Fawn to pale cinnamon; zonate and slightly sulcate	White to grayish orange	—	Ellipsoid	3−4.2 × 2.5−3	Corner, 1987
*R. fibulatus*	*	China: Guangxi	Annual, resupinate	—	Cream to pale buff when fresh, pinkish buff when dry	—	Broadly ellipsoid to subglobose	3.9−4.3 × 3−3.5	Yuan and Dai, 2012
*R. grandisporus*	*	Brazil	Annual, pileate to laterally stipitate	Pale umber to date brown; concentrically zonate and sulcate	Dark buff to pale snuff brown	—	Globose	6−7	Gomes-Silva et al., 2014
*R. hypobrunneides*	*	Malaysia	Annual, resupinate	—	Drab brown	Cylindric to subventricose, thin-walled hymenial cystidia, smooth	Pip-shaped	4−5.5 × 3−3.5	Corner, 1987
*R. incarnatus*	*	Sumatra	Annual, resupinate to effused-reflexed	Dark rubiginous baybrown; minutely velutinate-sulcate	Deep pinkish buff to pale cinnamon	Ventricose, slightly thick-walled hyphoid cystidia with crystals	Broadly ellipsoid to subglobose	2.5−3.5 × 2−2.5	Corner, 1987
*R. incurvus*	*	Malaysia	Laterally stipitate	Ochraceous to gray; zonate	Pale reddish brown, probably more pinkish when fresh	Hyphoid cystidia, smooth	Globose	3−4	Ryvarden, 1988
*R. laetus*	*	Australia: Victoria	Annual, pileate	Orange or orange-rufous; radially striate	Orange or orange-rufous to deep brick red	—	Subglobose to globose	4.5−6	Cooke, 1883
*R. malayanus*	*	Malaysia	Annual, effused-reflexed	Pallid cream, yellowish to isabelline; azonate	Pallid cream, yellowish to isabelline	—	Ellipsoid to subcylindric	5−6 × 3−3.5	Hattori, 2003
*R. mariae*	*	Brazil	Annual, pileate to laterally stipitate	Cinnamon, fulvous to snuff brown; concentrically zonate	Fulvous	—	Globose	4−6	Gomes-Silva et al., 2014
*R. micropendulus*	*	Ecuador	Annual, pendant with a distinct central to lateral stipe	Pale pinkish to beige; azonate	White to cream and staining reddish when fresh	Thick-walled hyphoid cystidia with crystals	Globose	3.5−4	Læssøe and Ryvarden, 2010
*R. mutabilis*	*	Costa Rica	Annual, pileate to laterally stipitate	White, pale gray, or brown when fresh, dark gray to reddish brown when dry; radially striate	White, peach colored to saffron when fresh, ochraceous to pale straw- colored when dry	—	Globose	3−4	Lindblad and Ryvarden, 1999
*R. nevadensis*	*	Venezuela	Annual, effused-reflexed	Ochraceous; azonate	Cream to pale orange	Thick-walled hyphoid cystidia with crystals	Ellipsoid	3−4 × 2.4−2.7	Ryvarden and Iturriaga, 2010
*R. ochraceicinctus*	*	Malaysia	Annual to perennial, resupinate	—	White when fresh, ochraceous to wood-colored when dry	—	Subglobose to globose	4.5−6	Ryvarden and Johansen, 1980; Corner, 1992
*R. parvulus*	*	Papua New Guinea	Annual, effused-reflexed	Pale ochraceous; faintly zonate	Pale ochraceous	Ventricose acute to acuminate, thin-walled hyphoid cystidia, smooth	Ovoid to globose	5−6.5 × 5−6	Corner, 1987
*R. patellarius*	*	Malaysia	Pendent with a very short stipe	Pale rufous ochraceous; wrinkled and slightly sulcate	Pale cream	Thick-walled hyphoid cystidia with crystals	Subglobose	5−6.5 × 4.5−6	Corner, 1987
*R. pellicula*	*	Indonesia	Annual, resupinate	—	Cream, ochraceous to pale brown	Thick-walled hyphoid cystidia with crystals	Broadly ellipsoid	4.5−6.5 × 3−4.5	Teixeira, 1992
*R. pendulus*	*	Indonesia: Sulawesi	Annual, pileate	Pale ochraceous to black; concentrically zonate and tuberculate	violaceous when fresh, black when dry	Thick-walled hyphoid cystidia with crystals	Globose	4−4.5	Ryvarden, 1990
*R. perennis*	*	Cameroon	Perennial, pileate	Dark brown; concentrically zonate and sulcate	Ochraceous when fresh, pale brown when dry	—	Globose	3−4	Ryvarden, 2019
*R. ravidus*	*	Russia	Annual, pileate	White, cream to pinkish buff; zonate	Cream to pale pinkish buff	Hyphoid cystidia with crystals	*	*	Pouzar, 1966
*R. subpileatus*	*	Malaysia	Annual, resupinate to effused-reflexed	Orange-cinnamon; wrinkled	White to cream	—	Subglobose	4.5−5.7	Corner, 1987
*R. substereinus*	*	Cuba	Pileate	Latericeous to bay; zonate and radially wrinkled	White to pallid white	—	Broadly ellipsoid to subglobose	2−3 × 2−2.5	Murrill, 1907
*R. subvinctus*	*	Zimbabwe	Annual, resupinate	—	Whitish gray	Club-shaped hyphoid cystidia with crystals; mammillate hymenial cystidia, smooth	Ellipsoid	3 × 2	Ryvarden, 2020
*R. tomentosus*	*	Zambia	Annual, pileate	Ochraceous; slightly concentrically zonate	Wood-colored	Thick-walled hyphoid cystidia with crystals	Globose	5−6	Ryvarden, 2018
*R. trametoides*	*	Solomon Islands	Annual, pileate	Fawn drab to fuscous blackish; zonate and sulcate	White then pale fuscous ochraceous	Thick-walled hyphoid cystidia with crystals	Subglobose	3.5−4.5 × 3.4	Corner, 1987
*R. umbonatipes*	*	Argentina	Annual, pileate to stipitate	Beige when fresh, light chestnut to grayish when dry; azonate	White when fresh, slightly pinkish red when dry	—	Subglobose to globose	4−5 × 4−4.5	Rajchenberg, 1987
*R. vinaceus*	*	Malaysia	Annual, pileate to stipitate	Light cinnamon vinaceous to pinkish flesh when fresh, dingy vinaceous when dry; faintly zonate and distinctly sulcate or wrinkled	Pinkish orange to brownish orange with a white bloom	Ventricose, hymenial cystidia, smooth	Pip-shaped to subglobse	3−3.7 × 2.5−3	Hattori, 2001b

Bold = new taxa. + = data available, — = Absent, * = data unavailable.

**Figure 2:**
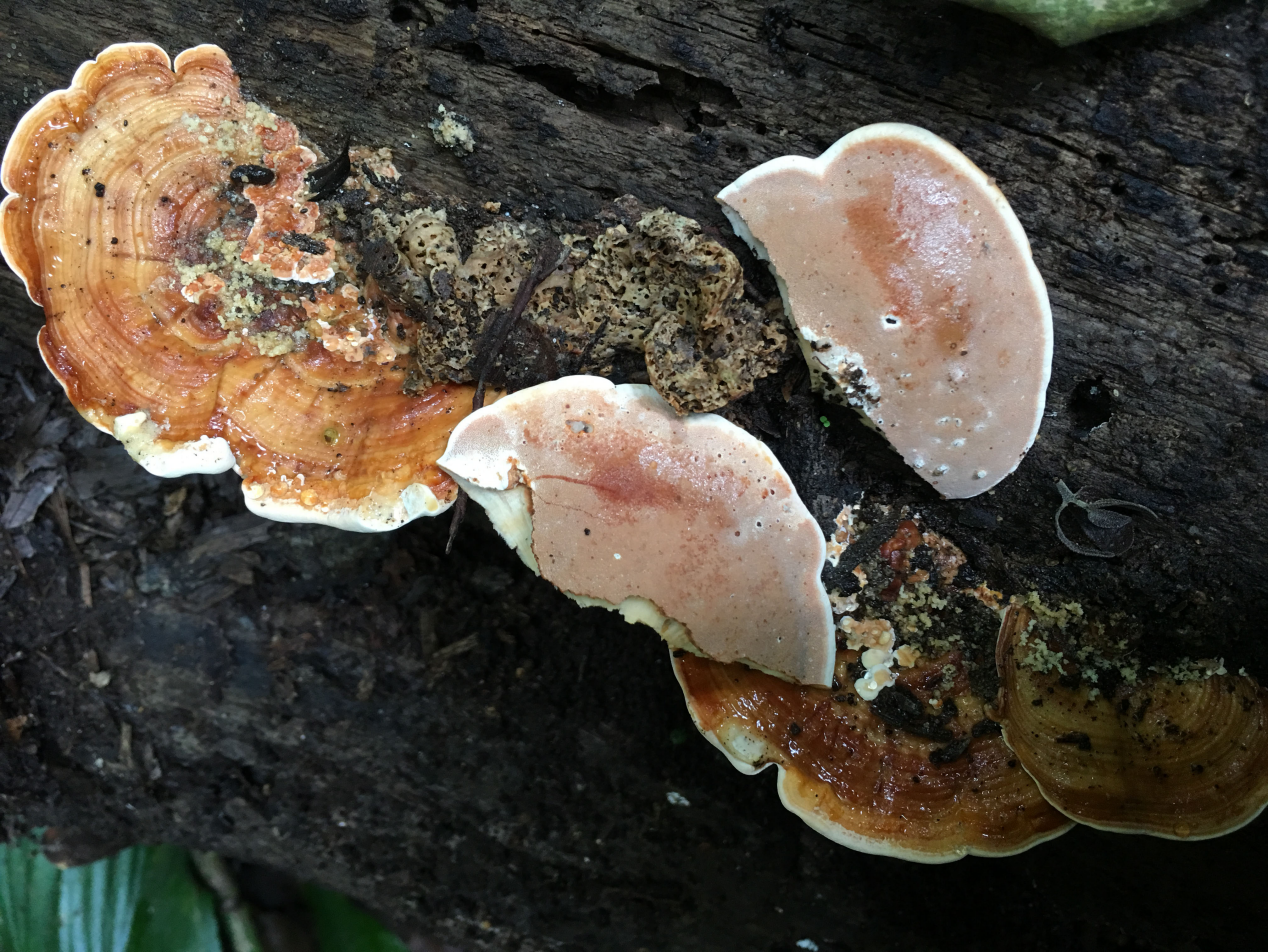
Basidiome of *Rigidoporus imbricatus* (holotype, Dai 17515).

**Figure 3:**
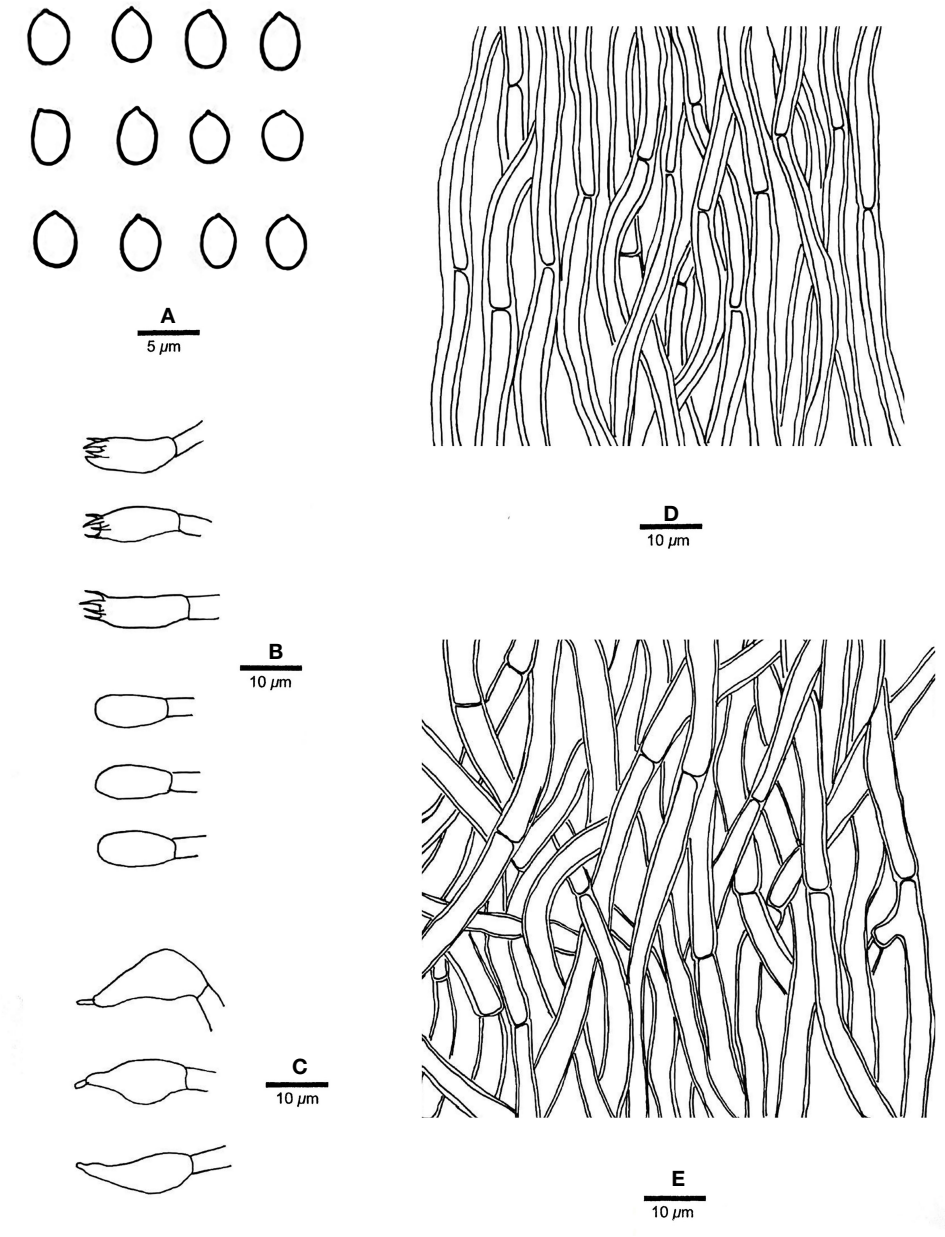
Microscopic structures of *R. imbricatus* (drawn from the holotype, Dai 17515). **(A)** Basidiospores; **(B)** Basidia and basidioles; **(C)** Cystidioles; **(D)** Hyphae from trama; **(E)** Hyphae from context. — Scale bars: a = 5 µm; b−e = 10 µm.

***Rigidoporus imbricatus*** Chao G. Wang, Jing Si & Y.C. Dai, sp. *nov.* — MycoBank: MB848605; **Figures 2**, **3**.

*Etymology*. — *Imbricatus* (Lat.), refers to the species having distinct imbricate basidiomata.

*Holotype*. — China. Yunnan Province, Mengla County, Wangtianshu Nature Reserve, on fallen angiosperm trunk, 18.VI.2017, Dai 17515 (BJFC025047).

*Additional specimen examined*. — Malaysia, Selangor, Kota Damansara, Community Forest Reserve, on dead angiosperm tree, 7.XII.2019, Dai 21180 (BJFC032834).

*Fruiting body*. — Basidiomata annual, effused-reflexed to pileate, corky, without odor or taste when fresh, becoming hard corky upon drying, up to 5 cm long, 4 cm wide when resupinate; pilei applanate to flabelliform, projecting up to 6 cm, 12 cm wide and 14 mm thick at base. Pileal surface yellowish brown to brownish orange when fresh, cinnamon to fawn, glabrous, concentrically zonate and sulcate, sometimes tuberculate when dry; margin blunt. Pore surface clay pink to flesh pink when fresh, becoming orange brown upon bruising, eventually honey-yellow to grayish brown when dry; sterile margin distinct, white when fresh, buff yellow when dry, up to 2.5 mm wide; pores round to angular, 7−9 per mm; dissepiments thin, entire to slightly lacerate. Context buff, corky when dry, up to 6 mm thick. Tubes stratified, cream near the context part, concolorous with pore surface near the pores part, hard corky when dry, up to 8 mm long.

*Hyphal structure*. — Hyphal system monomitic; generative hyphae simple septate, hyaline to yellowish, smooth, IKI−, CB−; tissues becoming black in KOH.

*Context.* — Contextual hyphae thick-walled with a wide lumen, rarely branched, rarely simple septate, straight to slightly flexuous, interwoven, 4−6 µm in diam.

*Tubes.* — Tramal hyphae distinctly thick-walled with a wide lumen, unbranched, straight to slightly flexuous, subparallel along the tubes, strongly agglutinated, 3−5.5 µm in diam. Cystidia absent; cystidioles ventricose with a pointed tip, thin-walled, smooth, 15−17 × 5−8 µm; basidia broadly clavate to barrel-shaped, bearing four sterigmata and a simple basal septum, 12−13 × 5−6 µm; basidioles of similar shape to basidia, but smaller.

*Spores.* — Basidiospores broadly ellipsoid to subglobose, hyaline, thin-walled, smooth, IKI−, CB−, (3.2−)3.4−4 × (2.7−)2.8−3.2(−3.5) µm, L = 3.64 µm, W = 3.05 µm, Q = 1.17−1.21 (n = 60/2).

*Notes*. — *R. imbricatus* is characterized by annual and imbricate basidiomata, concentrically zonate and sulcate upper surface, clay pink to flesh pink pore surface when fresh, ventricose cystidioles, broadly ellipsoid to subglobose, thin-walled basidiospores measuring 3.4−4 × 2.8−3.2 µm, and occurrence in tropical Asia.

*R. imbricatus*, *R. aurantiacus* Ryvarden & Iturr., and *R. pterocaryae* are phylogenetically related (**Figure 1**) but different in morphology. *R. aurantiacus* has deep-orange to ochraceous pore surface, globose basidiospores measuring 3 × 4 µm, and occurrence in South America (Ryvarden and Iturriaga, 2003). *R. pterocaryae* has azonate and smooth upper surface and larger thick-walled basidiospores (6.8−7.2 × 5.8−6.8 µm *vs.* 3.4−4 × 2.8−3.2 µm).

*R, dimiticus* (Corner) T. Hatt., *R. malayanus* (Corner) T. Hatt., and *R. pendulus* Ryvarden were originally described from Southeast Asia and have effused-reflexed to pileate or stipitate basidiomata. However, *R. dimiticus* has a dimitic hyphal system and larger basidiospores (4−4.7 × 3.5−4 µm *vs.* 3.4−4 × 2.8−3.2 µm; Hattori, 2001a); *R. malayanus* has effused-reflexed basidiomata with azonate upper surface, whitish pore surface, angular pores of 1−3 per mm and larger basidiospores (5−6 × 3−3.5 µm *vs.* 3.4−4 × 2.8−3.2 µm; Hattori, 2003); *R. pendulus* has violaceous pore surface when fresh, thick-walled hyphoid cystidia encrusted with crystals and globose basidiospores (3.4−4 µm *vs.* 3.4−4 × 2.8−3.2 µm; Ryvarden, 1990). Thus, they are all different from *R. imbricatus*.

**Figure 4:**
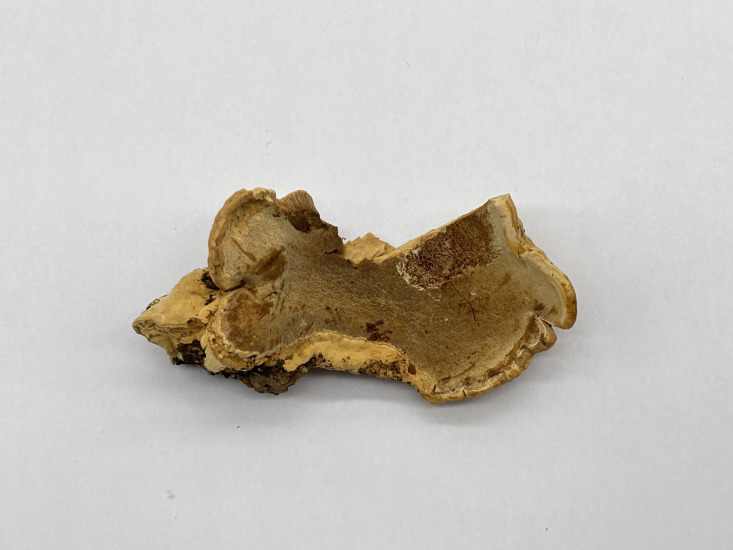
Basidiome of *R. pterocaryae* (holotype, Cui 4195).

**Figure 5:**
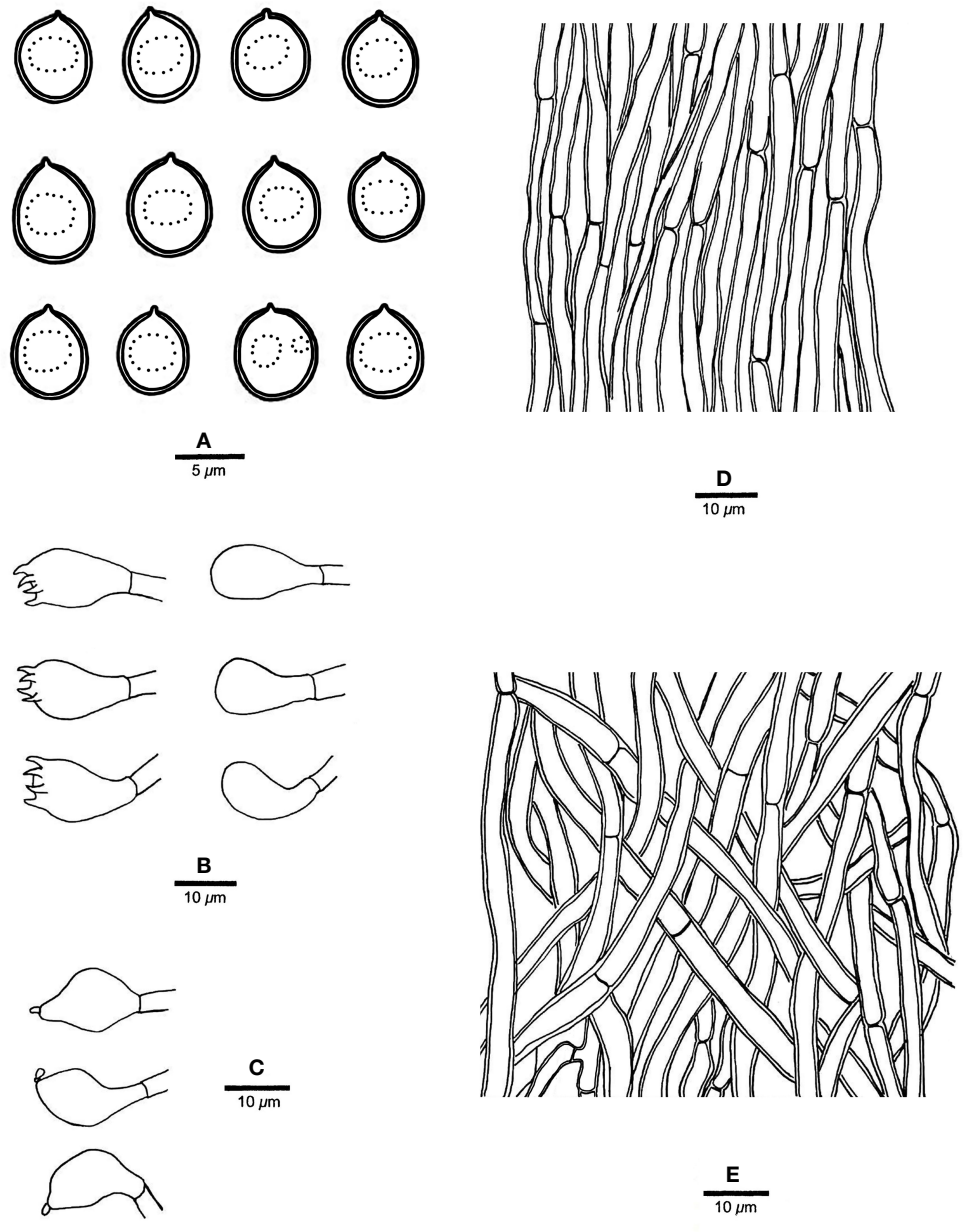
Microscopic structures of *R. pterocaryae* (drawn from the holotype, Cui 4195). **(A)** Basidiospores; **(B)** basidia and basidioles; **(C)** cystidioles; **(D)** hyphae from trama; and **(E)** hyphae from context. — Scale bars: a = 5 µm; b−e = 10 µm.

***Rigidoporus pterocaryae*** Chao G. Wang, Jing Si & Y.C. Dai, sp. *nov.* — MycoBank: MB848606; **Figures 4**, **5**.

*Etymology*. — *pterocaryae* (Lat.), refers to the species growth on *Pterocarya* sp.

*Holotype*. — China. Fujian Province, Wuyishan, Wuyi Palace, on living tree of *Pterocarya*, 29.VIII.2006, Cui 4195 (BJFC002245).

*Fruiting body*. — Basidiomata annual, pileate, corky, without odor or taste when fresh, becoming hard corky upon drying. Pilei petaloid, projecting up to 6 cm, 6.5 cm wide and 9 mm thick at base. Pileal surface honey yellow, glabrous, azonate, tuberculate when dry; margin irregularly lobed to slightly petaloid, blunt, slightly recurved when dry. Pore surface buff, honey yellow to fawn when dry, shiny; sterile margin distinct, cream when dry, up to 2.5 mm wide; pores round to angular, 7−9 per mm; dissepiments thin, slightly lacerate. Context curry yellow, corky when dry, up to 4 mm thick. Tubes concolorous with pore surface, hard corky when dry, up to 5 mm long.

*Hyphal structure*. — Hyphal system monomitic; generative hyphae simple septate, hyaline to yellowish, smooth, IKI−, CB+; tissues becoming black in KOH.

*Context.* — Contextual hyphae slightly thick- to thick-walled with a wide lumen, rarely branched, moderately simple septate, straight to flexuous, interwoven, 3−5 µm in diam.

*Tubes.* — Tramal hyphae slightly thick-walled with a wide lumen, unbranched, straight to slightly flexuous, subparallel along the tubes, agglutinated, 2.5−4 µm in diam. Cystidia absent; cystidioles ventricose with a pointed tip, thin-walled, smooth, 16−17 × 8−9 µm; basidia broadly barrel-shaped, bearing four sterigmata and a simple basal septum, 12−15 × 10−11 µm; basidioles of similar shape to basidia, but smaller. Rhomboid or irregular hyaline crystals present among hymenium.

*Spores.* — Basidiospores subglobose, hyaline, thick-walled, smooth, with one big or two small guttules, IKI−, CB−, (6.2−)6.8−7.5(−7.8) × (5.5−)5.8−7(−7.2) µm, L = 7.2 µm, W = 6.21 µm, Q = 1.15 (n = 30/1).

*Notes*. — *R. pterocaryae* is characterized by annual and pileate basidiomata, petaloid pilei, azonate and smooth upper surface, buff, honey yellow to fawn pore surface when dry, ventricose cystidioles, subglobose, thick-walled basidiospores measuring 6.8−7.5 × 5.8−7 µm, and growth on *Pterocarya* in China.

*R. pterocaryae* and *R. ulmarius* (Sowerby) Imazeki are similar in morphology, and share pileate basidiomata, azonate upper surface, subglobose, thick-walled, and almost the same size of basidiospores (this study). However, the latter has concentrically sulcate upper surface, and phylogenetically, they are distantly related (**Figure 1**).

One sequence of sample d1 from China was identified as *R. ulmarius* in GenBank (GenBank accession NO. KC414238). In our phylogenetic analysis, it nested together with *R. pterocaryae*.

**Figure 6:**
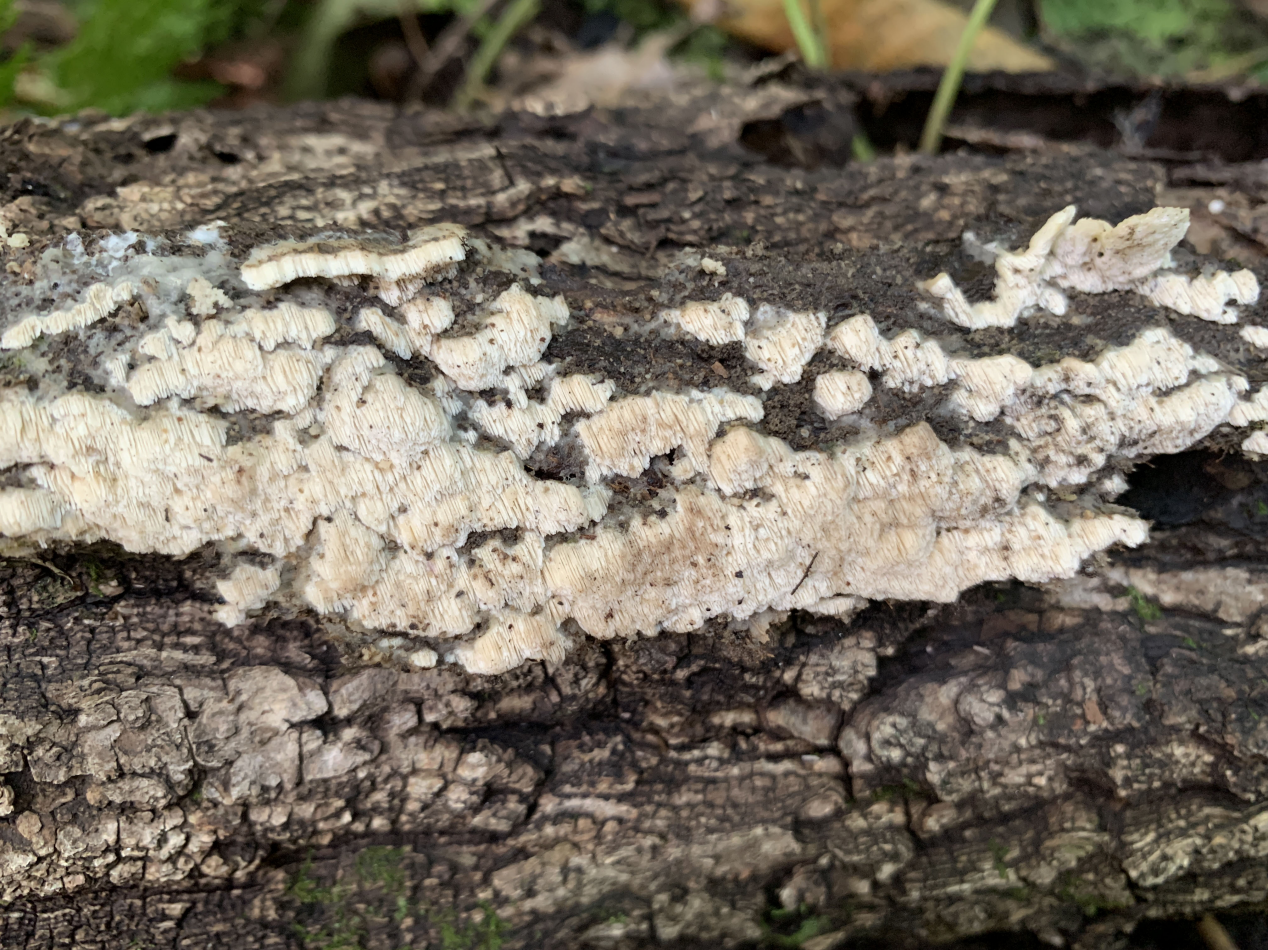
Basidiome of *R. subcorticola* (holotype, Dai 24419).

**Figure 7:**
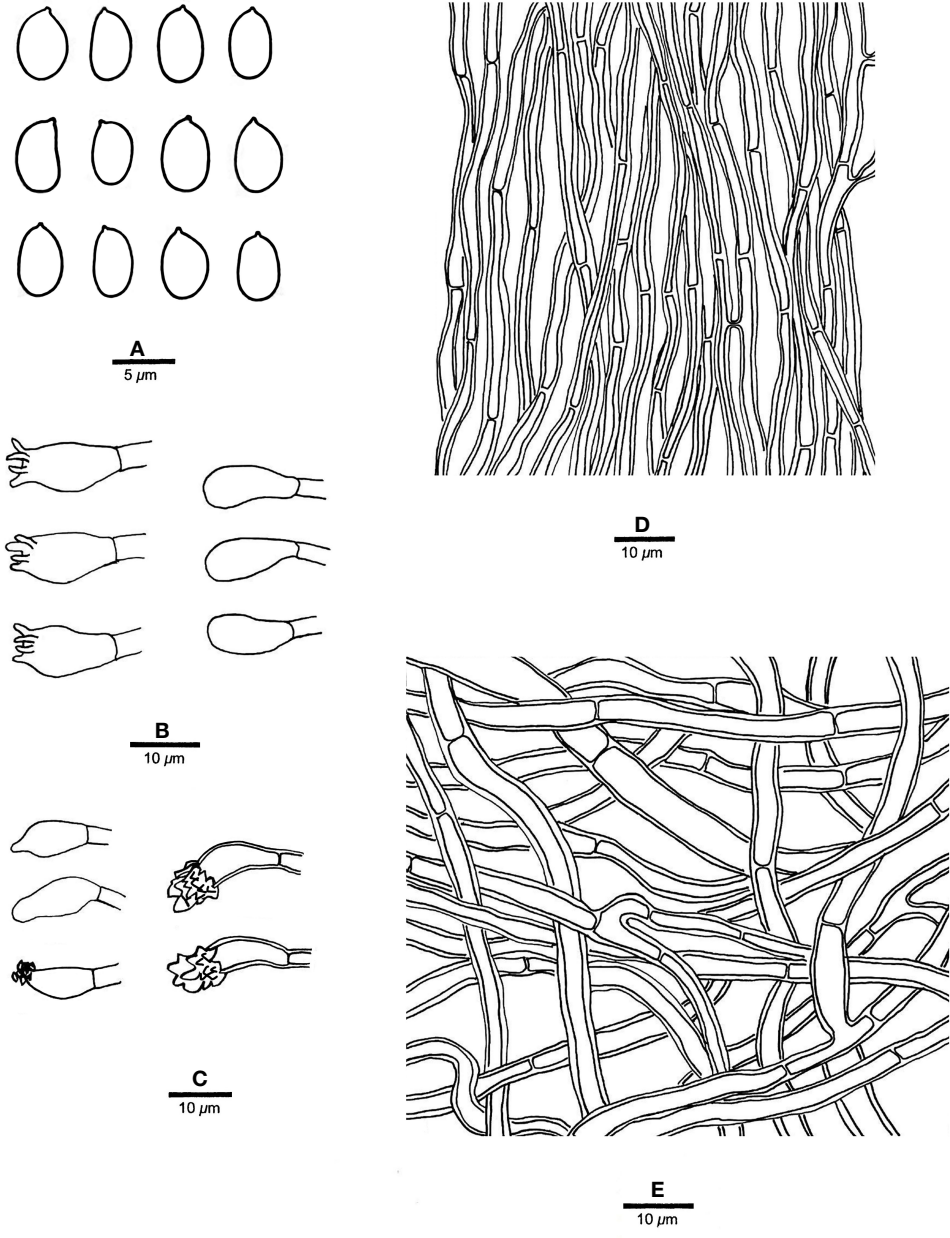
Microscopic structures of *R. subcorticola* (drawn from the holotype, Dai 24419). **(A)** Basidiospores; **(B)** basidia and basidioles; **(C)** cystidia and cystidioles; **(D)** hyphae from trama; and **(E)** hyphae from context. — Scale bars: a = 5 µm; b−e = 10 µm.

***Rigidoporus subcorticola*** Chao G. Wang, Jing Si & Y.C. Dai, sp. *nov.* — MycoBank: MB848607; **Figures 6**, **7**.

*Etymology*. — *subcorticola* (Lat.), refers to the species being similar to *R. corticola*.

*Holotype*. — China. Beijing, Xiaolongmen Forest Park, on fallen angiosperm trunk, 30.VIII.2022, Dai 24419 (BJFC039662).

*Additional specimens examined*. — China. Heilongjiang Province, Ning’an County, Jingpo Lake Forest Park, on *Pinus koraiensis*, Dai 8895 (IFP 011351); Henan Province, Neixiang County, Baotianman Nature Reserve, on *Juglans* sp., 23.IX.2009, Dai 11319 (BJFC007465).

*Fruiting body*. — Basidiomata annual, resupinate to effused-reflexed, soft, without odor or taste when fresh, becoming soft corky upon drying, up to 8 cm long and 2 cm wide when resupinate; pilei flabelliform, projecting up to 1.7 cm, 3 cm wide, 3 mm thick at base. Pileal surface white to cream when fresh, becoming cream to pinkish buff, glabrous, azonate and tuberculate upon drying; margin slightly acute. Pore surface white to cream when fresh, becoming cream to buff yellow upon drying; sterile margin white to cream when fresh, buff when dry, thinning out, somewhat incised, up to 1 mm wide; pores angular, 2−4 per mm; dissepiments thin, lacerate. Context cream, soft corky when dry, up to 0.4 mm thick. Tubes concolorous with pore surface, soft corky when dry, up to 0.8 mm long.

*Hyphal structure*. — Hyphal system monomitic; generative hyphae simple septate, hyaline, IKI−, CB+; tissues unchanged in KOH.

*Context.* — Contextual hyphae thick-walled with a wide lumen, occasionally branched, straight to flexuous, interwoven, 3−6 µm in diam.

*Tubes.* — Tramal hyphae thick-walled with a wide lumen, occasionally branched, straight to flexuous, subparallel along the tubes, 2−3.5 µm in diam. Cystidia present, arising from tramal hyphae and projecting from the hymenium, thick-walled, apically encrusted with crown crystals, 15−16 × 4.5−5 µm; cystidioles fusoid to ventricose, thin-walled, smooth or encrusted with tiny crystals, 12−15 × 5−6 µm; basidia barrel-shaped, with four sterigmata and a simple basal septum, 14−16 × 5.5−7 µm; basidioles of similar shape to basidia.

*Spores.* — Basidiospores oblong ellipsoid to ellipsoid, hyaline, thin-walled, smooth, IKI−, weakly CB+, 5−5.8(−6) × 3−4 µm, L = 5.44 µm, W = 3.48 µm, Q = 1.56 (n = 30/1).

*Notes*. — *R. subcorticola* is characterized by annual, resupinate to effused-reflexed basidiomata, azonate and tuberculate upper surface, cream to buff yellow pore surface when dry, big angular pores of 2−4 per mm, thick-walled and encrusted hymenial cystidia, fusoid to ventricose cystidioles, oblong ellipsoid to ellipsoid basidiospores measuring 5−5.8 × 3−4 µm, and often occur in the north temperate zone.

*R. subcorticola* and *R. macroporus* (Y.C. Dai & Y.L. Wei) F. Wu et al. are closely related in our phylogeny (**Figure 1**), share cream to buff yellow pore surface, clavate to ventricose cystidioles, and ellipsoid basidiospores, as well as are distributed in China. However, the later has resupinate basidiomata, thin-walled tramal hyphae and larger basidiospores (7−8 × 3.5−4.1 µm *vs.* 5−5.8 × 3−4 µm; Dai et al., 2004). *R. subcorticola* and *R. corticola* (Fr.) Pouzarare are very similar in morphology and share resupinate to effused-reflexed basidiomata with light-colored pore surface and ellipsoid basidiospores (5−6 × 3.5−4.5 µm *vs.* 5−5.8 × 3−4 µm; Ryvarden and Gilbertson, 1994). However, the latter has slightly zonate and somewhat radially wrinkled upper surface. The three species form different independent lineages in *Rigidoporus* (**Figure 1**).

**Figure 8:**
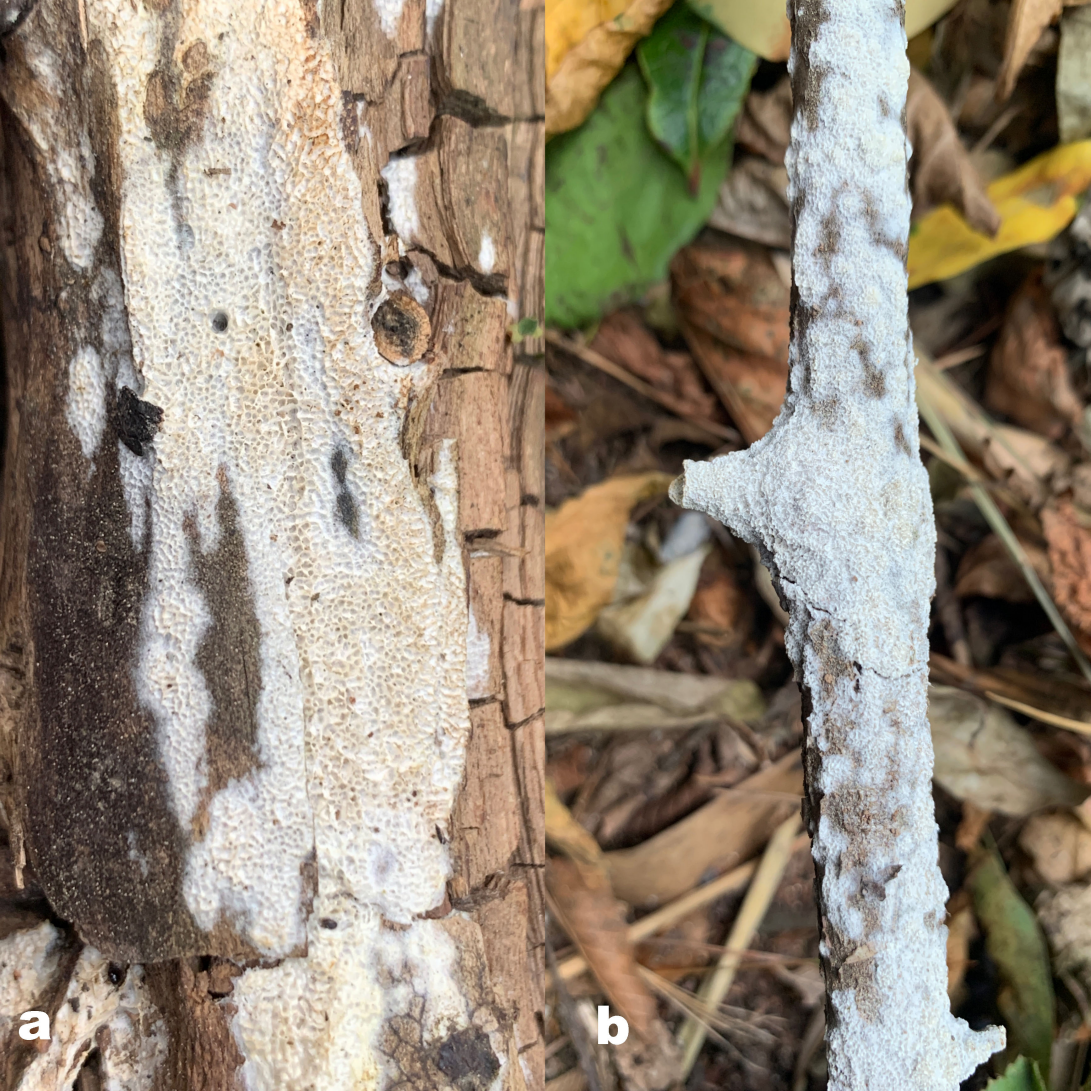
Basidiomata of two species: **(A)** *R. millavensis* (Dai 24509) and **(B)** *R. philadelphi* (Dai 24218).

***Rigidoporus millavensis*** (Bourdot & Galzin) Chao G. Wang, Jing Si & Y.C. Dai, *comb. et stat. nov.* — MycoBank: MB848608; **Figure 8**.

*Basionym*. — *Poria mucida* subsp. *millavensis* Bourdot & Galzin, Bull. trimest. Soc. mycol. Fr. 41(2): 238 (1925)

≡ *Oxyporus millavensis* (Bourdot & Galzin) Ryvarden & Melo, Syn. Fung. (Oslo) 31: 293 (2014).

*Specimens examined*. — China. Gansu Province, Zhangye, Qilianshan Nature Reserve, on the dead tree of *Juniperus przewalskii*, Dai 18970 (BJFC027439); Inner Mongolia, Alxa County, Helanshan Nature Reserve, on the fallen branch of *Picea*, Dai 24509 (BJFC039751); on the fallen trunk of *Picea*, Dai 24503 (BJFC039745); Xinjiang, Xinyuan County, Nalati Nature Reserve, on the fallen trunk of *Populus euphratica*, Wei 1622 (BJFC010351).

**Descriptions for one known species and one uncertain species**

**Figure 9:**
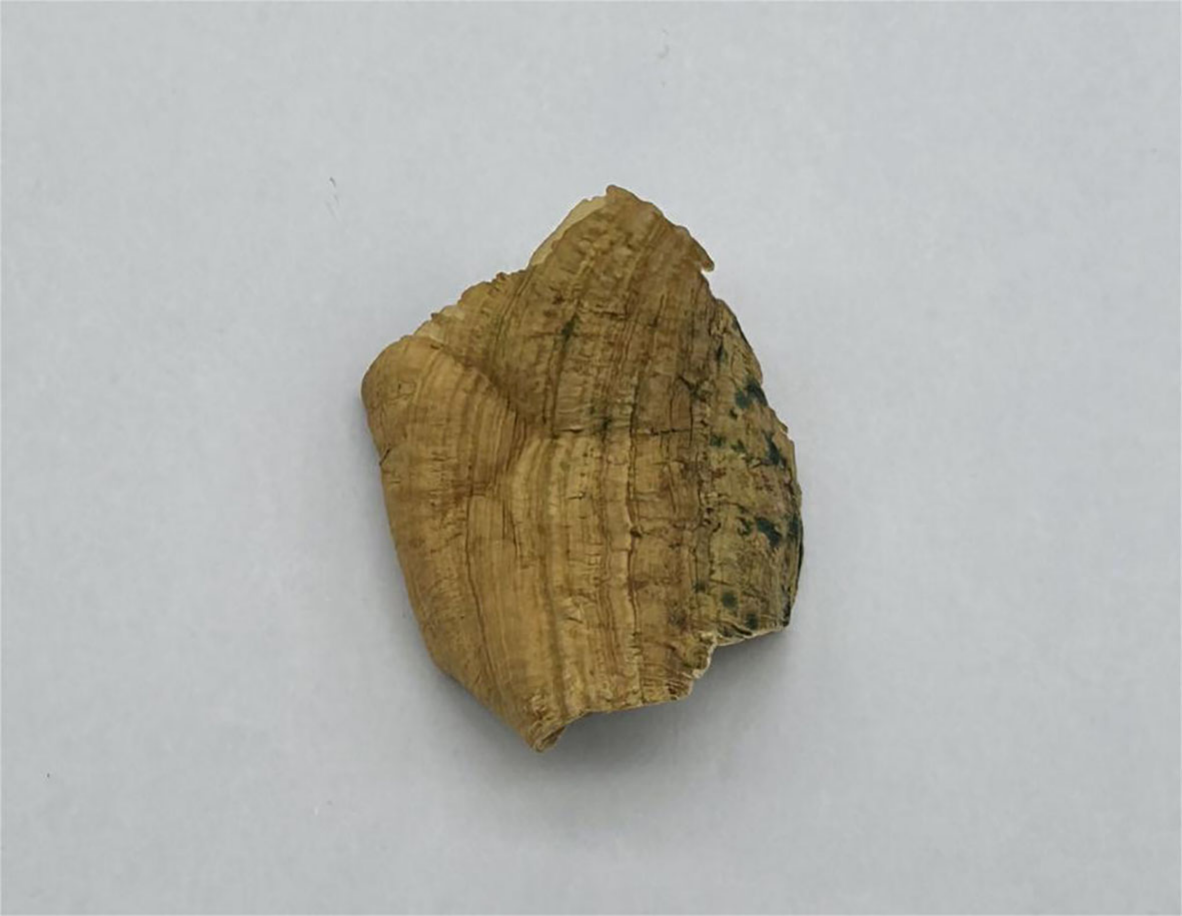
Basidiome of *R. microporus* (Dai 17402).

**Figure 10:**
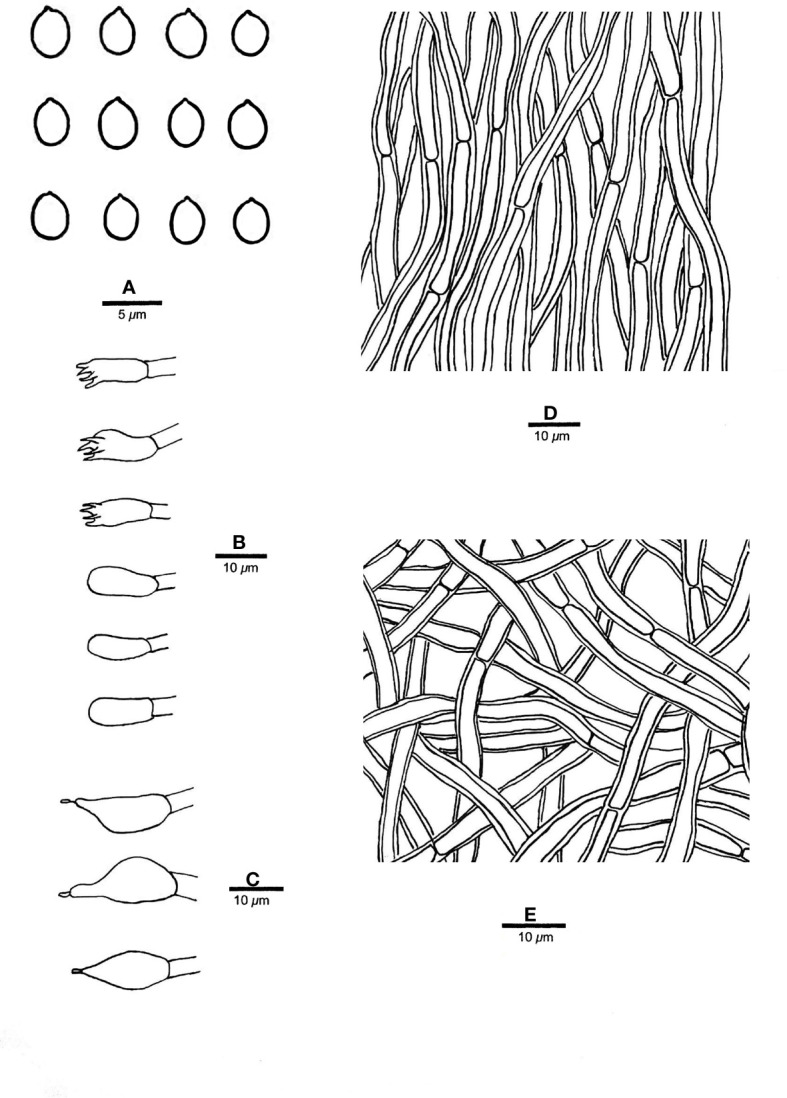
Microscopic structures of *R. microporus* (drawn from sample Dai 17402). **(A)** Basidiospores; **(B)** basidia and basidioles; **(C)** cystidioles; **(D)** hyphae from trama; and **(E)** hyphae from context. — Scale bars: a = 5 µm; b−e = 10 µm.

***Rigidoporus microporus*** — **Figures 9** and **10**

*Specimens examined*. — Brazil. Manaus, parque Municipal Cachoeira das Orquídeas, on a fallen angiosperm trunk, 12.V.2017, Dai 17402 (BJFC024937); Dai 17392 (BJFC024928).

*Fruiting body*. — Basidiomata annual to perennial, resupinate, effused-reflexed to pileate, corky, without odor or taste when fresh, becoming hard corky upon drying, up to 6 cm long, 5 cm wide when resupinate; pilei flabelliform, projecting up to 6 cm, 7.5 cm wide and 4 mm thick at base. Pileal surface cinnamon to fawn, glabrous, concentrically zonate and sulcate when dry; margin acute. Pore surface fawn to reddish brown when dry; sterile margin thinning out, pinkish buff when dry; pores round to angular, 12−14 per mm; dissepiments thin, entire to lacerate. Context buff, corky when dry, up to 1 mm thick. Tubes honey yellow to fawn when dry, paler than pore surface, hard corky when dry, up to 3 mm long.

*Hyphal structure*. — Hyphal system monomitic; generative hyphae simple septate, hyaline, smooth, IKI−, weakly CB+; tissues becoming black in KOH.

*Context.* — Contextual hyphae thick-walled with a wide lumen, unbranched, rarely simple septate, straight to slightly flexuous, interwoven, 3.5−5.5 µm in diam.

*Tubes.* — Tramal hyphae distinctly thick-walled with a wide lumen, unbranched, straight to slightly flexuous, subparallel along the tubes, strongly agglutinated, 3.5−5 µm in diam. Cystidia absent; cystidioles ventricose with a pointed tip, thin-walled, smooth, 10−15 × 7−8 µm; basidia broadly clavate to barrel-shaped, bearing four sterigmata and a simple basal septum, 10−12 × 4.5−6 µm; basidioles of similar shape to basidia, but smaller. Rhomboid or irregular crystals sometimes present among hymenium.

*Spores.* — Basidiospores broadly ellipsoid to subglobose, hyaline, thin-walled, smooth, IKI−, CB−, (3.6−)3.8−4.3 × (3−)3.2−3.8(−4) µm, L = 3.98 µm, W = 3.42 µm, Q = 1.16−1.17 (n = 60/2).

**Figure 11:**
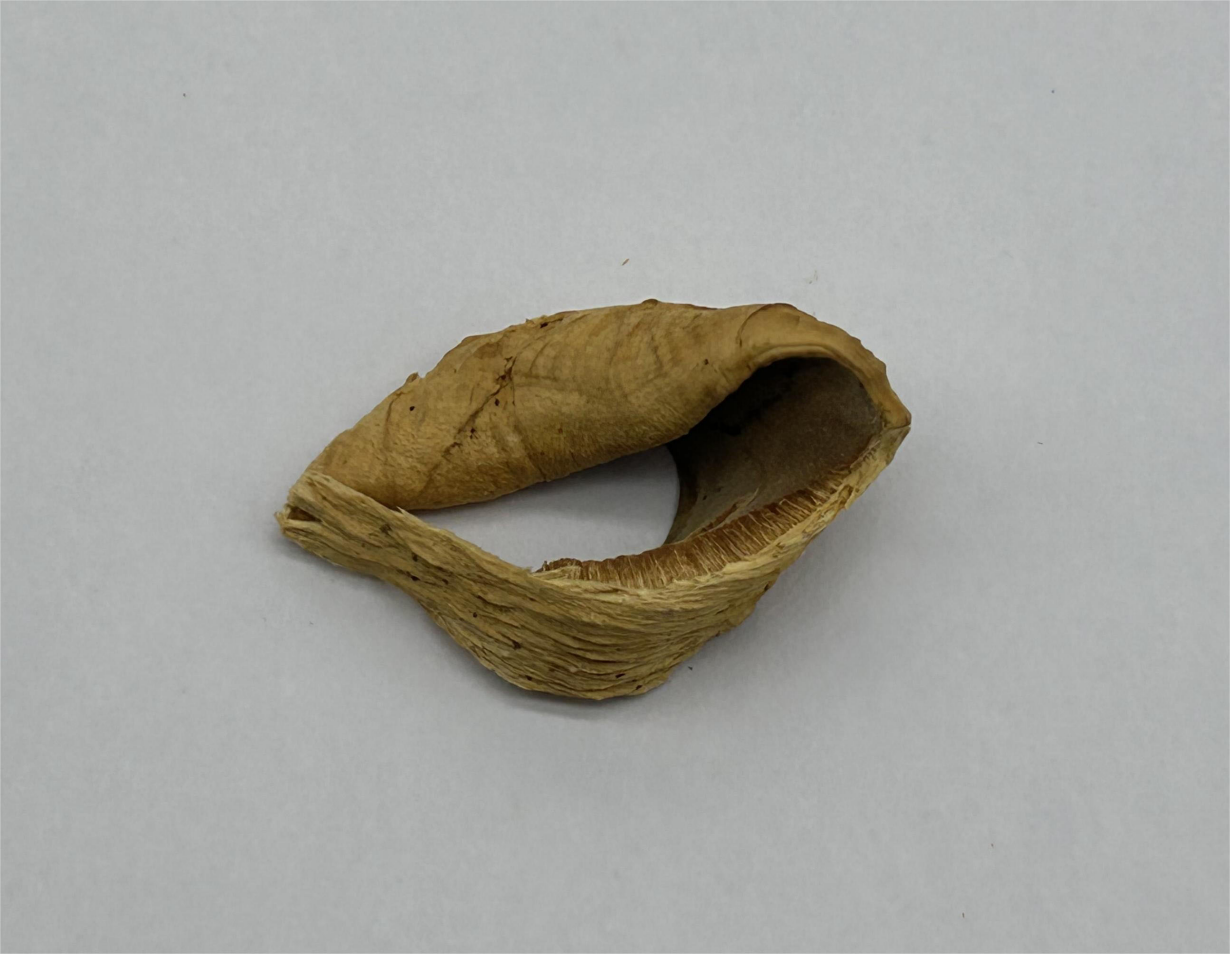
Basidiome of “*Rigidoporus ulmarius*” (JV 1909/17-J).

**Figure 12:**
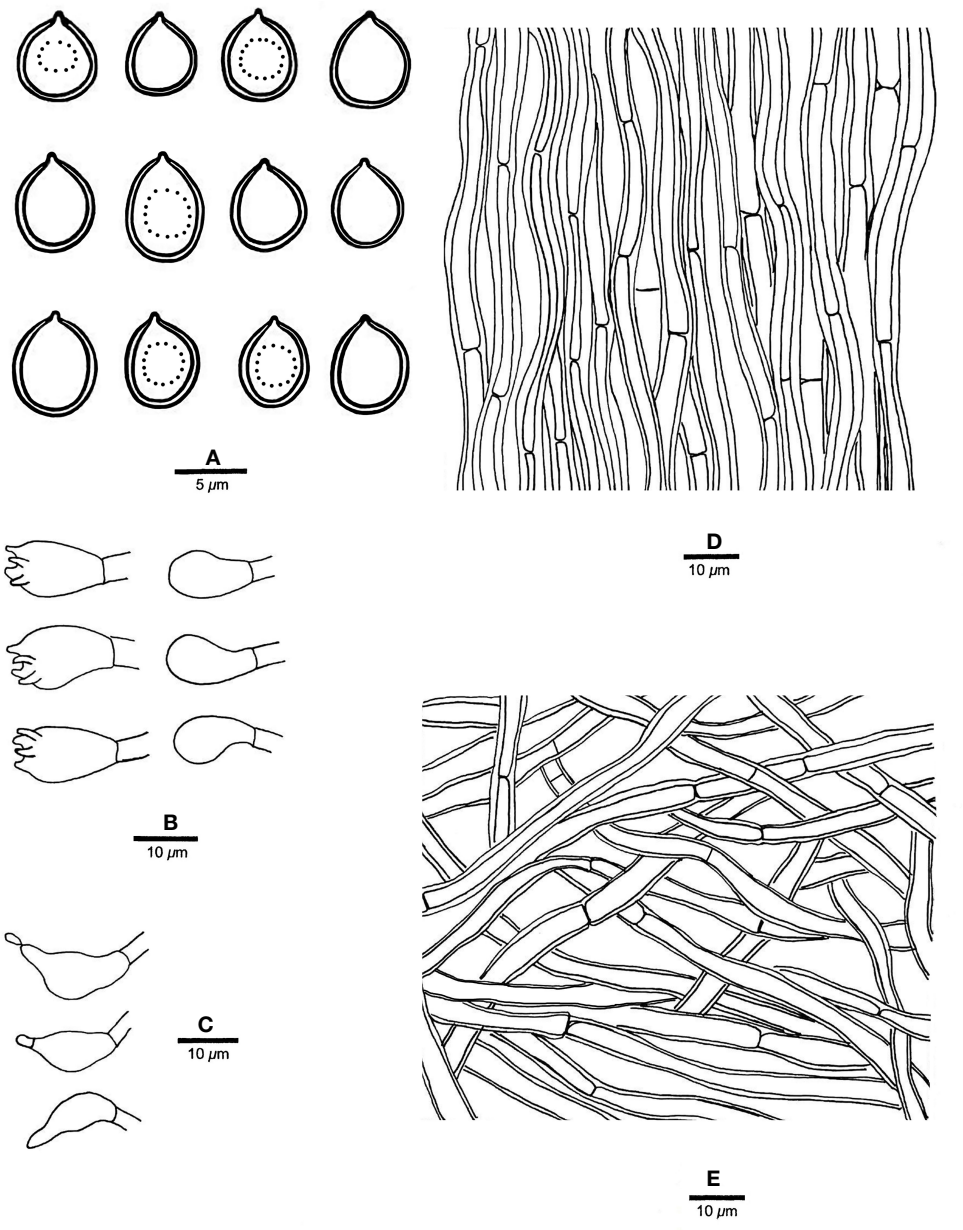
Microscopic structures of “*Rigidoporus ulmarius*” (drawn from sample JV 1909/17-J). **(A)** Basidiospores; **(B)** basidia and basidioles; **(C)** cystidioles; **(D)** hyphae from trama; and **(E)** hyphae from context. — Scale bars: a = 5 µm; b−e = 10 µm.

**“*Rigidoporus ulmarius*”** — **Figures 11** and **12**

*Specimen examined*. — French Guiana. Roura, Amazon Lodge, 2.IX.2019, JV 1909/17-J (JV, isotype in BJFC033010).

*Fruiting body*. — Basidiomata annual, pileate, corky, without odor or taste when fresh, becoming hard corky upon drying. Pilei flabelliform, projecting up to 8 cm, 15 cm wide and 7 mm thick at base. Pileal surface buff to cinnamon buff, glabrous, azonate, distinctly radially wrinkled to ribbed and slightly squamose when dry; margin acute, incurved when dry. Pore surface clay buff to fawn when dry; sterile margin indistinct, almost absent; pores angular, 8−10 per mm; dissepiments thin, slightly lacerate. Context ochraceous, corky when dry, up to 3 mm thick. Tubes concolorous with pore surface, distinctly deeper-colored than the context, hard corky when dry, up to 4 mm long.

*Hyphal structure*. — Hyphal system monomitic; generative hyphae simple septate, yellowish, smooth, IKI−, CB−; tissues unchanged in KOH.

*Context.* — Contextual hyphae thick-walled with a wide lumen, unbranched, moderately simple septate, more or less flexuous, interwoven, 3−6 µm in diam.

*Tubes.* — Tramal hyphae distinctly thick-walled with a wide lumen, unbranched, flexuous, subparallel along the tubes, 3.5−6 µm in diam. Cystidia absent; cystidioles ventricose with a pointed tip, thin-walled, smooth, 14−17 × 6−8 µm; basidia broadly barrel-shaped, bearing four sterigmata and a simple basal septum, 15−17 × 9−10 µm; basidioles of similar shape to basidia but smaller. Rhomboid or irregular hyaline crystals present among hymenium.

*Spores.* — Basidiospores subglobose, sometimes collapsed, hyaline, slightly thick-walled, smooth, sometimes with one big guttule, IKI−, CB+, (5.5−)6−7 × 5−6.2(−6.3) µm, L = 6.36 µm, W = 5.66 µm, Q = 1.12 (n = 30/1).

## 4 Discussion

In the present study, 18 species of *Rigidoporus* with available sequences were divided into four clades. Clade I includes six species, viz., *R. aurantiacus* Ryvarden & Iturriaga, *R. imbricatus*, *R. microporus*, *R. pterocaryae*, *R. submicroporus* F. Wu et al., and *R. ulmarius* (**Figure 1**), and these species share pileate and hard corky basidiomata with azonate, tuberculate or concentrically zonate, and sulcate upper surface (except *R. submicroporus*), slightly dark-colored (cinnamon to fawn or reddish brown) pore surface when dry, and sometimes thick-walled basidiospores. *R. corticola*, *R. ginkgonis* (Y.C. Dai) F. Wu et al., *R. macroporus*, *R. millavensis*, *R. philadelphi* (Parmasto) Pouzar, and *R. subcorticola* form a subclade as Clade II (**Figure 1**), and all have resupinate or resupinate to effused-reflexed, soft corky basidiomata with light-colored (white to cream or buff) pore surface. Clade III is composed of *R. obducens* (Pers.) Pouzar, *R. piceicola* (B.K. Cui & Y.C. Dai) F. Wu et al., *R. populinus* (Schumach.) Pouzar, and *R. subpopulinus* (B.K. Cui & Y.C. Dai) F. Wu et al. (**Figure 1**), and species in this clade have resupinate or resupinate to effused-reflexed basidiomata and thick-walled cystidia encrusted with coarse crystals. Clade IV contains two taxa, i.e., *R. cuneatus* (Murrill) F. Wu et al. and *R. juniperinus* Gafforov et al., but these two species are not similar in morphology. *R. cuneatus* has resupinate to effused-reflexed basidiomata with white to isabelline pore surface, angular to irregular pores of 1−3 per mm, thin-walled hymenial cystidia encrusted with crystals, and globose basidiospores measuring 3−5 µm (Yuan et al., 2020). *R. juniperinus* has resupinate basidiomata with white to pale ochraceous pore surface, round to angular pores of 4−5 per mm, thin-walled hymenial cystidia encrusted with crystals, thick-walled encrusted hyphoid cystidia, and broadly ellipsoid to globose basidiospores measuring 4.2−4.5 × 2.9−3 µm (Yuan et al., 2020). Clades I, II, and III above represent traditional genera *Rigidoporus* (s. str.), *Chaetoporus* P. Karst., and *Oxyporus* Donk, which were however united in *Rigidoporus* by Wu et al. (2017).

*Boletus ulmarius* Sowerby was originally described by Sowerby (1797) growing on rotted *Ulmus campestris* from the UK and then combined as *R. ulmarius* by Imazeki (1952). It is characterized by perennial, pileate basidiomata with concentrically sulcate or irregularly tuberculate upper surface, pinkish buff to orange brown pore surface when fresh, angular pores of 5−6 per mm, a monomitic hyphal system, fusoid cystidioles, subglobose and thick-walled basidiospores measuring 6−8 × 5−6.5 µm, and mostly growth on angiosperm wood in the north temperate zone (Gerber and Loguercio-Leite, 1997; Ryvarden and Melo, 2017). The Chinese sample Dai 18490A nests together with two British samples KM 178999 and KM 155306 and Hawaiian sample JV 2211/H3-J, forming an independent lineage with a robust support, and is treated as *R. ulmarius* in our phylogeny (**Figure 1**). Morphologically, the Chinese sample Dai 18490A also has pileate basidiomata, slightly zonate, concentrically sulcate and radially wrinkled upper surface, round to angular pores of 5−7 per mm, and subglobose, thick-walled basidiospores measuring 5.5−7 × 5−6.5 µm. The Chinese sample Cui 4195 and American sample JV1909/17-J are very similar with *R. ulmarius* by pileate basidiomata with azonate upper surface, and subglobose, thick-walled and almost the same size of basidiospores (6.8−7.5 × 5.8−7 µm in Cui 4195, 6−7 × 5−6.2 µm in JV1909/17-J). However, they have smaller pores (7−9 per mm in Cui 4195, 8−10 per mm in JV1909/17-J, *vs.* 5−6 per mm in Dai 18490A) and form respectively independent lineages distinctive from Dai 18490A *etc*. *Polyporus cytisinus* Berk., *Po. actinobolus* Mont., *Po. sublinguaeformis* Schulzer, *Po. geotropus* Cooke, *Placodes incanus* Quél., and *Po. fraxineus* Lloyd are listed as synonyms of *R. ulmarius* in Index Fungorum (http://www.indexfungorum.org/). Unfortunately, we did not study the types of these taxa, and for the time being, we treat the three American samples JV 1909/17-J, JV 1403/5-J, and JV 1504/40 as “*Rigidoporus ulmarius*” in our study.

*Boletus microporus* Sw. was originally described by Swartz (1788) from Jamaica. It is characterized by occasionally resupinate but mostly pileate basidiomata with concentrically zonate and sulcate upper surface, bright orange to reddish-brown pore surface, the absence of cystidia, ventricose smooth cystidiols, and broadly ellipsoid to subglobose basidiospores measuring 3.5−5 × 3.5−4 µm. Three America samples Dai 17402, Dai 17392, and JV 2110/1 form an independent lineage in *Rigidoporus*, and they also have completely resupinate or effused-reflexed to pileate basidiomata, concentrically zonate and sulcate upper surface, broadly ellipsoid to subglobose basidiospores measuring 3.8−4.3 × 3.2−3.8 µm, which is in accordance with the descriptions of *R. microporus*. In addition, four sequences of *R. microporus* (GenBank: KJ559458, KJ559468, AB697722, and HQ400709) deposited in GenBank form another lineage in our study, and they are from Africa (Nigeria and Cameroon) and Southeast Asia (Indonesia and Malaysia). Many synonyms of *R. microporus* are listed in Index Fungorum (http://www.indexfungorum.org/), and in order to avoid conflict with them, four Asian and African samples FRIM 646, taxon 219653, ED 310, and N 402 are regarded as “*Rigidoporus microporus*” in our study. *O. mollis* Gibertoni & Ryvarden, *R. amazonicus* Ryvarden, *R. grandisporus* Ryvarden et al., and *R. mariae* Gibertoni et al. were also originally described from South America (Brazil). However, *O. mollis* has bigger pores than three America samples (5–6 per mm *vs.* 12–14 per mm; Gibertoni et al., 2012), the other three species have stipitate basidiomata (Ryvarden, 1987; Gomes-Silva et al., 2014).

*O. millavensis* (Bourdot & Galzin) Ryvarden & Melo is morphologically similar and phylogenetically related to *R. philadelphi* (**Figure 1**). In addition, Ryvarden and Melo (2017) thought that they are synonymous species. *P. mucida* subsp. *millavensis* Bourdot & Galzin (1925) as the basionym of *O. millavensis* was originally described from France growth on rotting wood of *Juniperus* and *Pinus*. It also has resupinate basidiomata with grayish white to ochraceous pore surface, lacerate dissepiments, two types of smooth cystidia, and ovoid, broadly ellipsoid to subglobose basidiospores measuring 4.5−6.5 × 3.5−4.7 µm (Michel et al., 2005). *Chaetoporus philadelphi* Parmasto was originally described by Parmasto (1959) growth on the bark of *Philadelphus coronarius* from Estonia and then combined as *R. philadelphi* by Pouzar (1966). It is characterized by annual, resupinate basidiomata with white to pale-yellow pore surface, reticulated, angular pores of 2−3 per mm, encrusted or smooth cystidia, and broadly ellipsoid basidiospores measuring 4.5−5.5 × 3.8−4.5 µm (Parmasto, 1959). Four Chinese samples Dai 24503, Dai 24509, Dai 18970, and Wei 1622 and two Chinese samples Dai 24218 and Dai 24219 form two independent lineages nested in *Rigidoporus*, and all these six samples are very similar by resupinate basidiomata with cream, buff-yellow to gray pore surface, angular pores of 2−3 per mm, lacerate dissepiments, numerous small crystals, two type cystidia, and broadly ellipsoid and almost the same size of basidiospores (4.8−5.8 × 4−4.8 µm in the former four samples; 5.2−6.2 × 4.5−5.6 µm in the latter two samples), which is in accordance with the descriptions of *R. millavensis* and *R. philadelphi*. However, the former grows on both gymnosperm and angiosperm woods (*Juniperus*, *Picea*, and *Populus*), while the latter only grows on angiosperm wood. In addition, there are 14-base pair differences between them, which accounts for > 2% nucleotide differences in the ITS regions (610 bp in total). Thus, the four Chinese samples Dai 24503, Dai 24509, Dai 18970, and Wei 1622 are treated as *R. millavensis* and the two samples Dai 24218 and Dai 24219 are treated as *R. philadelphi* in this study.

Three new species of *Rigidoporus* are described in the present paper, and most of them are from tropical Asia. Most of these new species have rather similar morphology as existing species but formed independent lineages in phylogeny; thus, we deal with as new species. The same phenomenon was found in several other polypore genera (Yuan et al., 2021; Wang et al., 2022; Yuan et al., 2022; Si et al., 2023; Zhang et al., 2023; Zhou et al., 2023), and it seems that more new taxa will be found after further investigation and phylogenetic analyses.

## Data availability statement

The datasets presented in this study can be found in online repositories. The names of the repository/repositories and accession number(s) can be found below: https://www.ncbi.nlm.nih.gov/genbank/, OQ930240-OQ930284; OQ924520-OQ924549.

## Author contributions

C-GW, JV, CJ, and JS designed the research and contributed to data analysis and interpretation. C-GW and JV prepared the samples. C-GW, JV, and JS conducted the molecular experiments and analyzed the data. C-GW prepared the samples and drafted the manuscript. JV and JS discussed the results and edited the manuscript. All authors contributed to the article and approved the submitted version.
